# Neuropathic pain phenotyping by international consensus (NeuroPPIC) for genetic studies: a NeuPSIG systematic review, Delphi survey, and expert panel recommendations

**DOI:** 10.1097/j.pain.0000000000000335

**Published:** 2015-10-22

**Authors:** Oliver van Hecke, Peter R. Kamerman, Nadine Attal, Ralf Baron, Gyda Bjornsdottir, David L.H. Bennett, Michael I. Bennett, Didier Bouhassira, Luda Diatchenko, Roy Freeman, Rainer Freynhagen, Maija Haanpää, Troels S. Jensen, Srinivasa N. Raja, Andrew S.C. Rice, Ze'ev Seltzer, Thorgeir E. Thorgeirsson, David Yarnitsky, Blair H. Smith

**Affiliations:** a Division of Population Health Sciences, University of Dundee, Dundee, Scotland, United Kingdom; b Brain Function Research Group, School of Physiology, Faculty of Health Sciences, University of the Witwatersrand, Johannesburg, South Africa; c INSERM U-987, Centre d'Evaluation et de Traitement de la Douleur, CHU Ambroise Paré, Assistance Publique Hôpitaux de Paris, Boulogne-Billancourt, France; d Université Versailles Saint-Quentin, Versailles, France; e Division of Neurological Pain Research and Therapy, Department of Neurology, Universitätsklinikum Schleswig-Holstein, Kiel, Germany; f deCODE Genetics/Amgen, Reykjavík, Iceland; g Nuffield Department of Clinical Neurosciences, University of Oxford, Oxford, United Kingdom; h Academic Unit of Palliative Care, Leeds Institute of Health Sciences, University of Leeds, Leeds, United Kingdom; i The Alan Edwards Centre for Research on Pain, McGill University, Montreal, Canada; j Department of Neurology, Beth Israel Deaconess Medical Centre, Harvard Medical School, Boston, USA; k Zentrum für Anästhesiologie, Intensivmedizin, Schmerztherapie und Palliativmedizin, Benedictus Krankenhaus, Tutzing, Germany; l Klinik für Anästhesiologie, Technische Universität München, München, Germany; m Department of Neurosurgery, Helsinki University Central Hospital, Helsinki, Finland; n Mutual Insurance Company Etera, Helsinki, Finland; o Department of Clinical Medicine, Danish Pain Research Center, Aarhus University, Aarhus, Denmark; p Department of Neurology, Aarhus University Hospital, Aarhus, Denmark; q Department of Anesthesiology and Critical Care Medicine, Johns Hopkins University, School of Medicine, Baltimore, USA; r Pain Research, Department of Surgery and Cancer, Faculty of Medicine, Imperial College, Chelsea and Westminster Hospital campus, London, United Kingdom; s Pain Phenomics and Genomics Lab, University of Toronto Centre for the Study of Pain, University of Toronto, Toronto, Canada; t Department of Neurology, Technion Faculty of Medicine, Rambam Health Care Campus, Haifa, Israel (O. van Hecke is now with Nuffield Department of Primary Care Health Sciences, University of Oxford, Oxford, United Kingdom)

**Keywords:** Neuropathic pain, Genetics, Phenotype, Systematic review, Delphi survey

## Abstract

For genetic research to contribute more fully to furthering our knowledge of neuropathic pain, we require an agreed, valid, and feasible approach to phenotyping, to allow collaboration and replication in samples of sufficient size. Results from genetic studies on neuropathic pain have been inconsistent and have met with replication difficulties, in part because of differences in phenotypes used for case ascertainment. Because there is no consensus on the nature of these phenotypes, nor on the methods of collecting them, this study aimed to provide guidelines on collecting and reporting phenotypes in cases and controls for genetic studies. Consensus was achieved through a staged approach: (1) systematic literature review to identify all neuropathic pain phenotypes used in previous genetic studies; (2) Delphi survey to identify the most useful neuropathic pain phenotypes and their validity and feasibility; and (3) meeting of experts to reach consensus on the optimal phenotype(s) to be collected from patients with neuropathic pain for genetic studies. A basic “entry level” set of phenotypes was identified for any genetic study of neuropathic pain. This set identifies cases of “possible” neuropathic pain, and controls, and includes: (1) a validated symptom-based questionnaire to determine whether any pain is likely to be neuropathic; (2) body chart or checklist to identify whether the area of pain distribution is neuroanatomically logical; and (3) details of pain history (intensity, duration, any formal diagnosis). This NeuroPPIC “entry level” set of phenotypes can be expanded by more extensive and specific measures, as determined by scientific requirements and resource availability.

## Abstract 2

Supplemental Digital Content is Available in the Text.

NeuPSIG proposes a consensus-based standardised approach to phenotyping neuropathic pain for genetic studies in humans and guidelines for reporting the phenotyping of cases and controls.

## 1. Introduction

Genetic analysis of neuropathic pain will illuminate the biological processes underlying the condition and facilitate identification of novel therapeutic and prevention targets. However, the clinical utility of findings from current research is questionable, with limited success in identifying the genes contributing to the heritable risk, and difficulties in replicating the documented genes.^28,32^ Future genome-wide association and candidate gene studies will likely identify the genetic contribution to neuropathic pain heritability, as has happened with various complex central nervous system disorders, if sufficiently powered sample sizes are used.^17,41^ Although measures to ensure the quality and validity of genetic analysis are well established,^1,10,38^ there is little agreement on which neuropathic pain traits should be studied and which phenotyping tools to use. The quality and validity of the phenotype are as important as those of the genetic methodology,^50^ and perhaps even more important than sample size.^30^ A poorly defined or quantified phenotype will produce errors in effect size estimation, loss of power to identify risk genes, or identification of significant associations with phenotypes with limited clinical relevance.^24,33^

An optimally collected phenotype should fully describe the neuropathic pain entity under study every time and capture the same genetic variants in replicated studies. It should be collected with validated instruments (ie, accurate, precise, reproducible, with known positive and negative predictive values) that are practical (ie, simple to implement, efficient, cost-effective) and ethical. No single phenotyping instrument will encompass all these requirements. Rather, a number of instruments are required, ranging from “highly specific” tools to “very general.” These instruments should be mutually consistent and able to diagnose participants by classifying them as “possibly,” “probably”, or “definitely” having neuropathic pain.^47^ Classification of controls is as important as classification of cases, and both require stringency and clarity.^30^ Tailoring the right combination of instruments for any particular study depends on a balance between validity and feasibility.

There is currently no “gold standard” for assessing neuropathic pain, for clinical or research purposes. There is, however, growing consensus, with generally minor variations between approaches.^19,44^ Consensus on neuropathic pain phenotyping in genetic research will facilitate: (1) scientific collaboration, increasing the potential to combine cohorts to achieve larger sample sizes; (2) replication of gene discoveries; (3) meta-analyses; and (4) translation from laboratory to general population, and vice versa.^43^ Furthermore, until very large samples are created purely for researching neuropathic pain, population-based research will rely on data from multipurpose clinical samples. Although the lack of a “gold standard” currently precludes calculation of precise positive and negative predictive values, the existence of an agreed standard for neuropathic pain will facilitate a valid approach to this research.

Led and funded by the International Association for the Study of Pain (IASP) Special Interest Group (SIG) on Neuropathic Pain (NeuPSIG), and in collaboration with the IASP SIG on Genetics and Pain, we staged an approach towards achieving consensus on neuropathic pain phenotyping for human genetic studies. This is intended to inform future genetic research, rather than phenotyping for clinical research or clinical diagnosis.

## 2. Methods

Our step-wise approach towards this goal had 3 stages:

(1) A systematic literature review to identify all neuropathic pain phenotypes used in previous genetic studies;
(2) A Delphi survey to determine and rank neuropathic pain phenotypes assessing their validity and feasibility; and
(3) A meeting of experts to reach consensus on “ideal” phenotype(s) to be collected from patients with neuropathic pain for genetic studies.

### 2.1. Systematic review

#### 2.1.1. Aim

To conduct a systematic literature review to identify and compare phenotypes used in genetic studies of noncancer neuropathic pain in adults for the purposes of informing the phenotypes that may best capture the genetic architecture of neuropathic pain for human genetic studies.

#### 2.1.2. Study selection and data extraction

Electronic databases MEDLINE, EMBASE, SCOPUS, Science Direct, ISI Web of Science, and CINAHL were searched from January 1966 to April 2014 for English language papers. We used the same search strategy as a recent systematic review to identify neuropathic pain^48^ and combined this with broad search terms for “genetic studies.” These search terms are listed in Supplementary Digital Content 1 (available online as Supplemental Digital Content at http://links.lww.com/PAIN/A147).

We excluded studies in which: (1) it was not possible to distinguish between participants with neuropathic pain and those with nonneuropathic pain (including mixed pain conditions); (2) the pain was cancer related; (3) the condition was not currently defined by the IASP as neuropathic pain (complex regional pain syndrome, temporomandibular disorders, migraine, chronic widespread pain [CWP], and fibromyalgia)^26^; or (4) the study was in children.

Database searches were conducted by one author (O.v.H.). Article titles and then abstracts were reviewed for possible inclusion by 2 authors (O.v.H. and B.H.S.), before full-text versions of the remaining articles were reviewed and the final selection was confirmed. We extracted data on: (1) study characteristics (country, sample population, study design); (2) phenotyping methods described; (3) sample size (cases and controls); (4) the specific neuropathic pain condition(s) under study; and (5) genetic factors investigated.

We differentiated between “brief” and “detailed” phenotype descriptions to give an indication of the level of detail provided by the authors and whether or not it allowed replication by other researchers. For example, “Clinical examination by pain specialist”^15^ was regarded as “brief,” whereas “Clinical examination: straight leg raising test, manual testing of motor and sensory defects of the lower extremities concordant with magnetic resonance imaging findings,”^35^ was regarded as “detailed.” This does not necessarily indicate that only a brief examination was conducted in the former case, but indicates the extent to which the phenotyping was described.

In addition to extracting descriptive information, the phenotype information was assessed against the neuropathic pain grading guidelines published by NeuPSIG^47^ and approved by the IASP.^26^ This ranks the diagnostic certainty with which the presence or absence of neuropathic pain can be based on the neuroanatomical distribution of symptoms, patient history, and tests confirming the underlying nervous system lesion or disease. The grading system has the following criteria: (1) pain with a neuroanatomically plausible distribution; (2) a history of a relevant lesion or disease affecting the somatosensory system; (3) confirmatory tests demonstrating the presence of negative and positive sensory signs confined to innervation territory of the lesioned nervous structure; and (4) further diagnostic tests confirming a causative lesion or disease entity. Criteria 1 and 2 must be met to allow a working hypothesis of “possible” neuropathic pain. Additionally, criterion 3 or criterion 4 must also be met to reach the grade of “probable” neuropathic pain. If all 4 criteria are satisfied, the grade of “definite” neuropathic pain is achieved.

### 2.2. Delphi survey

#### 2.2.1. Aim

The aim was to obtain expert consensus on phenotype components that should be used to determine “caseness” in genetic studies of neuropathic pain and to grade the validity and feasibility of applying these phenotype components in research setting.

#### 2.2.2. Ethics approval

The study had ethics approval from the University of Dundee Research Ethics Committee (UREC 14032).

#### 2.2.3. Participants

E-mail invitations to take part in a 3-round Delphi survey were sent to 28 experts in the field of neuropathic pain phenotyping and/or conducting genetic studies on neuropathic pain. All experts were identified by their publication track record in at least one of the fields. The invitation provided information on the context and objectives of the Web-based survey (composed using the SurveyMonkey software application at https://www.surveymonkey.com) and a hyperlink for interested individuals to access the survey. Participation was voluntary, and anonymity was assured.

At the end of the first round of the survey, respondents indicated whether they wanted to take part in subsequent rounds. Respondents who elected to continue participating were sent e-mail invitations that included a summary of the results from the previous round, and a hyperlink to a new Web-based questionnaire. Round 3 was only completed after the face-to-face consensus meeting (see 2.3. Consensus meeting).

#### 2.2.4. Questionnaires

In round 1, expert panelists used a 5-point Likert scale (1 = strong disagreement, 3 = no agreement or disagreement, 5 = strong agreement) to rate the level of their agreement with statements regarding: (1) the sensitivity and specificity of symptoms, clinical signs, and additional investigations (eg, quantitative sensory testing [QST], nerve conduction studies) when diagnosing neuropathic pain; (2) the feasibility of nonexpert clinicians and researchers to accurately assess items in the 3 measurement domains (symptoms, clinical signs, and additional investigations); (3) whether symptoms and clinical signs could be self-assessed by study participants; and (4) whether the assessment of medical history, body charts of perceived pain, quality of life, and psychological factors should also be phenotyped in population-based genetic studies. The expert panelists were also asked to list up to 4 symptoms, signs, and additional investigations that they thought provided the best balance between feasibility and validity when assessing whether a pain was predominantly neuropathic in nature. Finally, respondents rated the level of diagnostic certainty (none, possible, probable, or definite) they thought was achieved by 9 different assessment combinations. The 9 assessment combinations were: *symptoms only; symptoms, body chart of perceived pain and pain history; clinical signs only; clinical signs and symptoms; clinical signs, symptoms, body chart of perceived pain and pain history; additional investigations only; additional investigations and clinical signs; additional investigations, clinical signs, and symptoms; and additional investigations, clinical signs, symptoms, and body chart of perceived pain and pain history*. These assessments were derived from the neuropathic pain grading system developed by Treede and et al.^47^ The order of the 9 combinations was randomized for each panelist.

In round 2, following the standard Delphi methodology, to allow re-evaluation of responses in light of those of their peers,^13,39^ panelists were shown summary results from round 1. They were asked again for their responses to questions on measurement sensitivity, specificity, feasibility, whether participants could self-complete a diagnostic questionnaire, and on the resultant diagnostic certainty. Based on responses to round 1, panelists were also provided with a list of 14 verbal descriptors of their pain symptoms (*hot/burning, stabbing, itching, numbness, electric shocks/shooting, pricking/tingling/pins and needles, pain in an area of numbness, pain in a plausible anatomical distribution, pain evoked by light touch, pain in an area of altered sensation, spontaneous pain, evoked pain, painful cold, and paroxysmal pain*), 12 clinical signs (*dynamic mechanical allodynia, deep mechanical allodynia, altered sensation to punctate mechanical stimuli, static mechanical hyperalgesia, hypoesthesia to punctate mechanical stimuli, altered reflexes, punctate mechanical hyperalgesia, thermal hyperalgesia, cold allodynia, altered vibration sense, thermal hypoesthesia, and temporal summation*), and 5 additional investigations (*QST, cerebral evoked potentials, intraepidermal nerve fiber density, magnetic resonance imaging, and nerve conduction studies*). From each of these lists, panelists were asked to rank in descending order of importance the 5 items they thought provided the best balance between validity and feasibility when making a diagnosis of neuropathic pain. The order of items in each list was randomized for each panelist.

Finally, based on feedback from the first round, panelists were asked to identify up to 4 additional phenotype components that could be added to the assessment of study participants once “*caseness*” had been established, which would allow more complex phenotypes to be collected.

In round 3, respondents were shown summary results from round 2, and asked to rerate their ranking of the 5 most valid and feasible symptoms, clinical signs, and additional investigations to use when making a diagnosis of neuropathic pain. In addition, based on discussions at the face-to-face consensus meeting (see 2.3. Consensus meeting), which took place between round 2 and 3 of the Delphi survey, respondents were asked to rate their agreement on a 5-point Likert scale (1 = strong disagreement and 5 = strong agreement) with statements regarding the patient history and using a pain body chart. Because these additional questions were only asked once, responses were analyzed separately from the rest of the Delphi survey.

#### 2.2.5. Data analysis

Consensus in Delphi surveys is said to have been achieved when a given proportion of participants agree on an item under debate; this proportion varies between studies. For this study, achievement of “good” consensus was assumed when ≥70% of respondents agreed, and “strong” consensus was assumed when there was ≥90% agreement.^44^

### 2.3. Consensus meeting

#### 2.3.1. Aim

The aim was to develop a consensus statement on an approach to phenotyping in genetic studies of neuropathic pain in adults and to identify a basic or “entry level” phenotype for any such study.

#### 2.3.2. Procedure

The meeting was held in Versailles, France, from June 12 to 13, 2014, and consisted of 18 experts, identified by NeuPSIG based on their experience in the fields of pain phenotyping, epidemiology, and/or pain genetics. Represented disciplines included neurology, anaesthesiology, pain medicine, palliative care, primary care, basic neuroscience, and genetics. Activities on the first day included: (1) describing the aims of the meeting and defining the questions that were to be addressed; (2) presentation of data from the systematic literature review and the results of the first 2 rounds of the Delphi survey; and (3) short presentations by panelists on the differences and commonalities between “phenotyping” (what information to collect and how to collect it) to collect vs “phenomics” (which pain phenotypes should be used in genetic association analysis), phenotyping of complex diseases, phenotyping by questionnaires, phenotyping by clinical examination, phenotyping using standard and dynamic QST, and phenotyping for clinical trials. Each presentation by a panelist was followed by discussion. Activities on the second day included parallel breakaway discussions followed by plenary-based consensus. These focused on (1) the availability and use of validated diagnostic neuropathic pain screening tools, (2) the level of diagnostic certainty achieved when defining cases and controls, and (3) generating a consensus definition and the requirements of what should be “entry level” phenotyping requirements for genetic studies on neuropathic pain. All participants in the consensus meeting contributed as authors of this article.

## 3. Results

### 3.1. Systematic literature review

**Figure 1:**
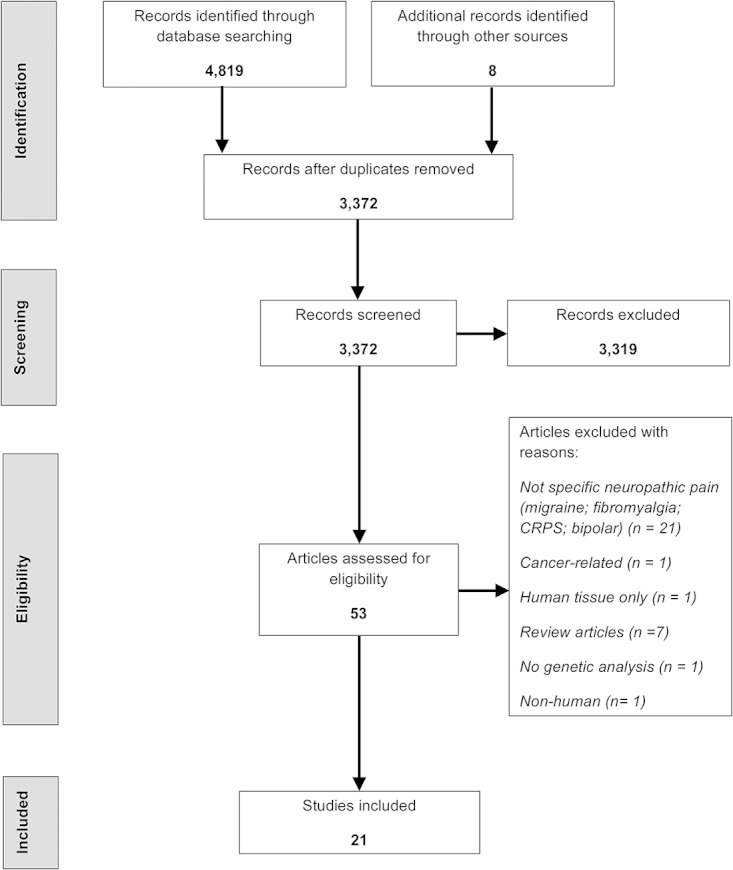
Systematic review: PRISMA flow diagram of article identification, assessment, and inclusion.

From an initial 4827 article titles identified through searching electronic databases and handsearching, 3372 were identified as unique records. Of these, 21 articles fulfilled the inclusion criteria and underwent data extraction (Fig. 1).

#### 3.1.2. Characteristics of included studies

Six studies analysed Nordic populations,^6,15,22,23,27,35^ 5 analysed other European populations,^2,4,16,20,40^ 3 analysed Japanese,^36,42,45^ 2 analysed Israeli-Jewish^12,34^ and South African black, Caucasian, and Indian,^21,49^ 1 analysed Chinese (Taiwan),^8^ and 1 analysed Caucasian-, African-, and Hispanic-American populations.^46^ One study included 5 cohorts with various neuropathic pain conditions from 4 populations (Danish, Finnish, Israeli-Jewish, and Caucasian-, African-, and Hispanic-American).^11^ Two studies on postherpetic neuralgia (PHN) recruited participants from the same Japanese population.^36,45^ Several studies reused the same cohorts to investigate different polymorphisms, namely 2 studies on a South African HIV-positive cohort comprising African black, Caucasian, and Indian,^21,49^ 2 studies on a Norwegian cohort with discogenic sciatic pain,^22,23^ 2 studies on Caucasian-, African-, and Hispanic-American patients with persistent pain after surgery for discogenic sciatic pain,^11,46^ and 3 studies on a Finnish cohort with discogenic pain.^11,27,35^

A variety of causes of neuropathic pain were studied (Table 1). Four study cohorts grouped together a number of causes of neuropathic pain,^2,4,11,15^ whereas others focused on a solitary cause of neuropathic pain.^6,8,12,16,20–23,27,34–36,40,42,45,46,49^ Overall, most associations reported between genotype and risk of neuropathic pain/intensity of neuropathic pain have been isolated findings that have not yet been replicated, or failed to replicate as conflicting findings have been reported. Although there have been consistent reports of genetic associations at specific loci, some of the replication studies included patients with postherpetic neuralgia recruited from the same study site^36,45^ or from the same study cohort^27,35^ (ie, patients having discogenic sciatic pain) (Table 2). Only 3 studies included a priori replication cohorts into their study designs.^11,15,46^

**Table 1:**
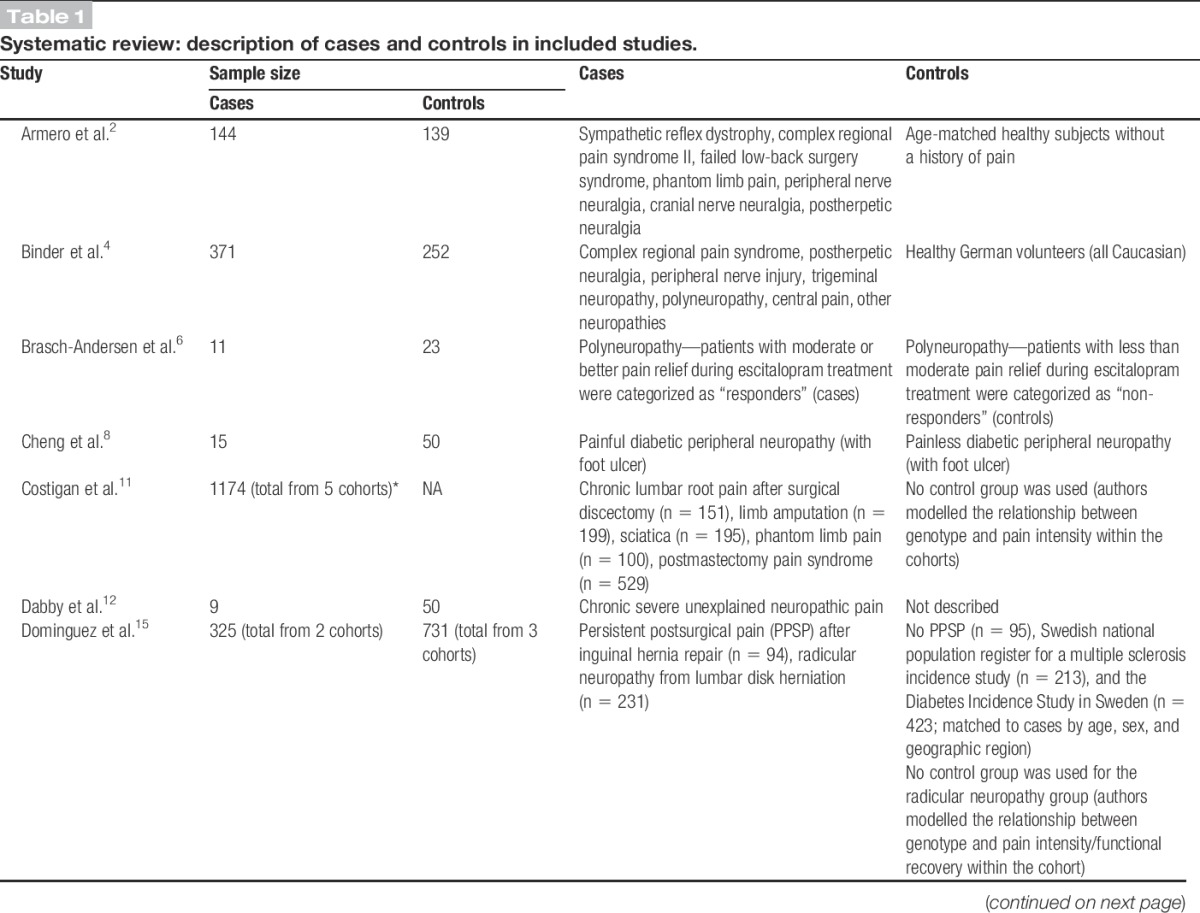

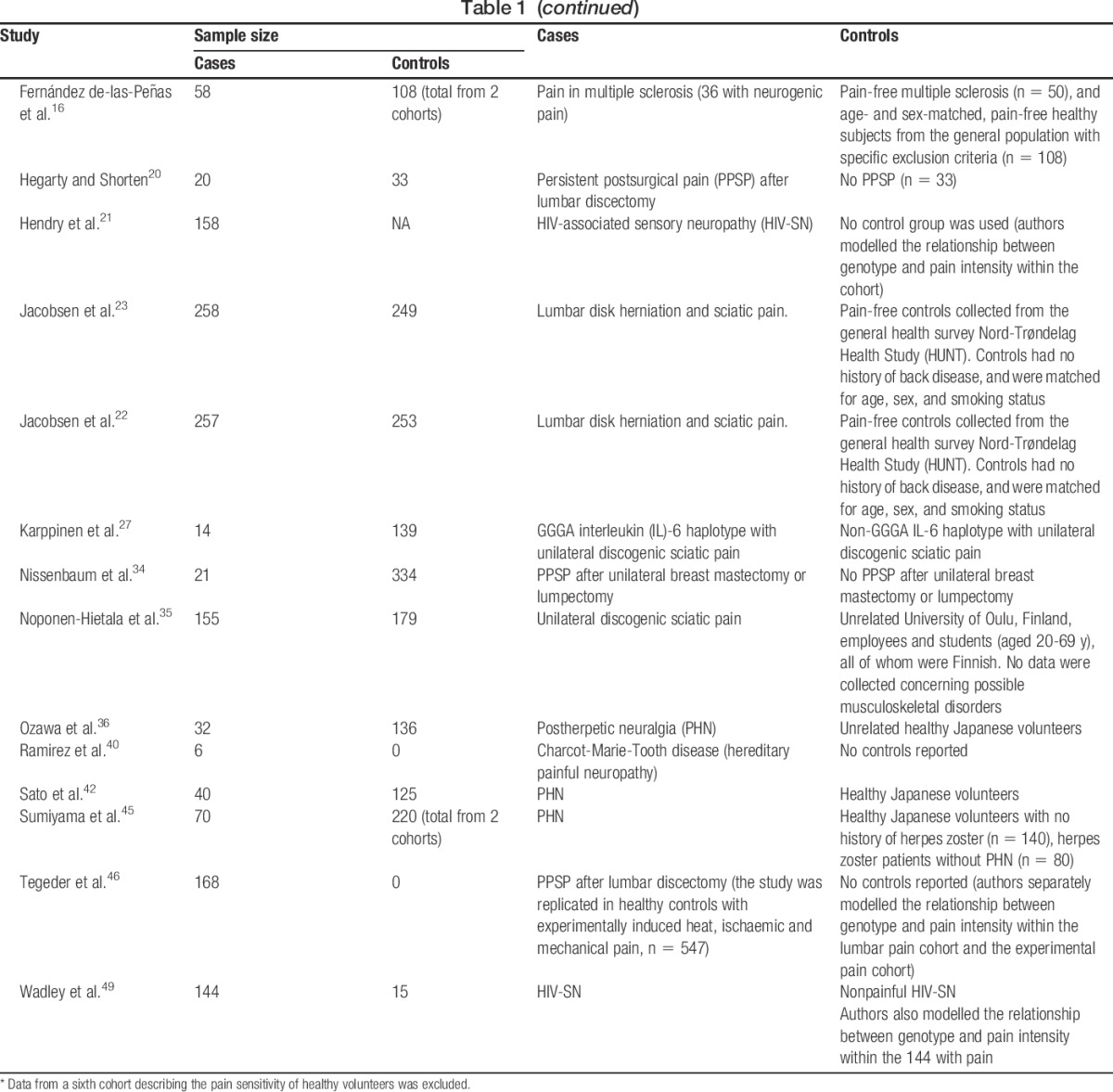
Systematic review: description of cases and controls in included studies.

**Table 2:**
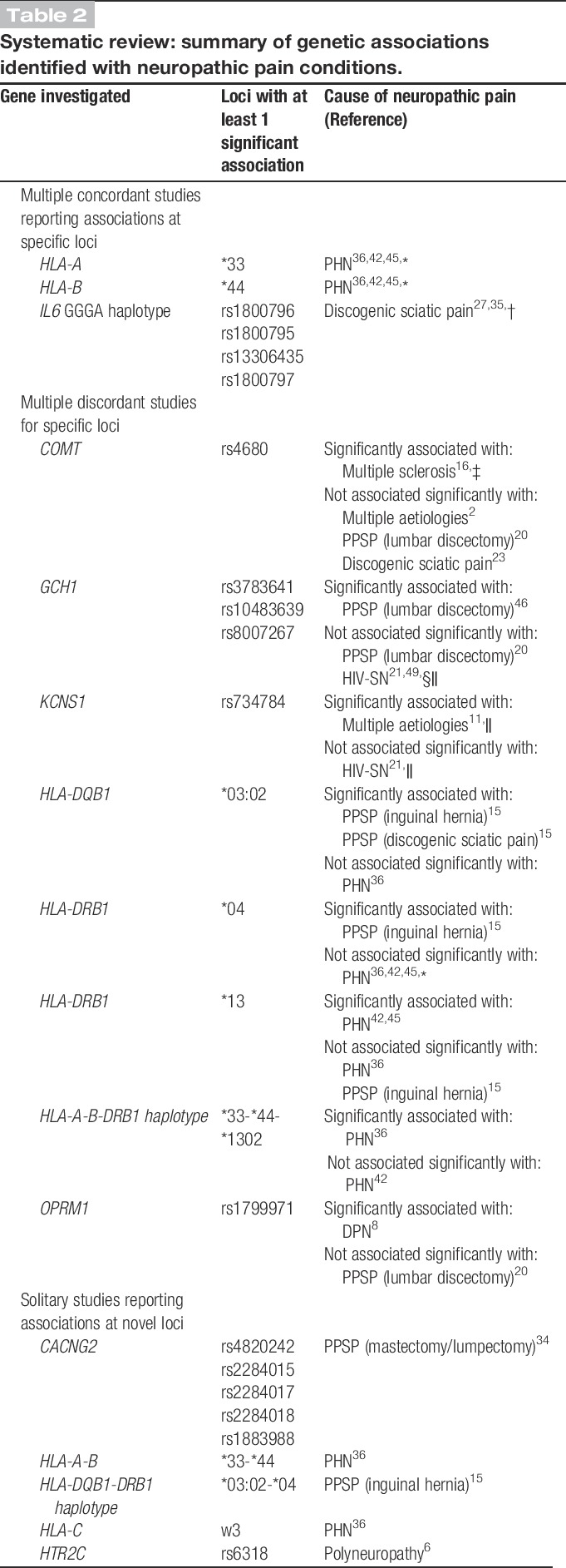

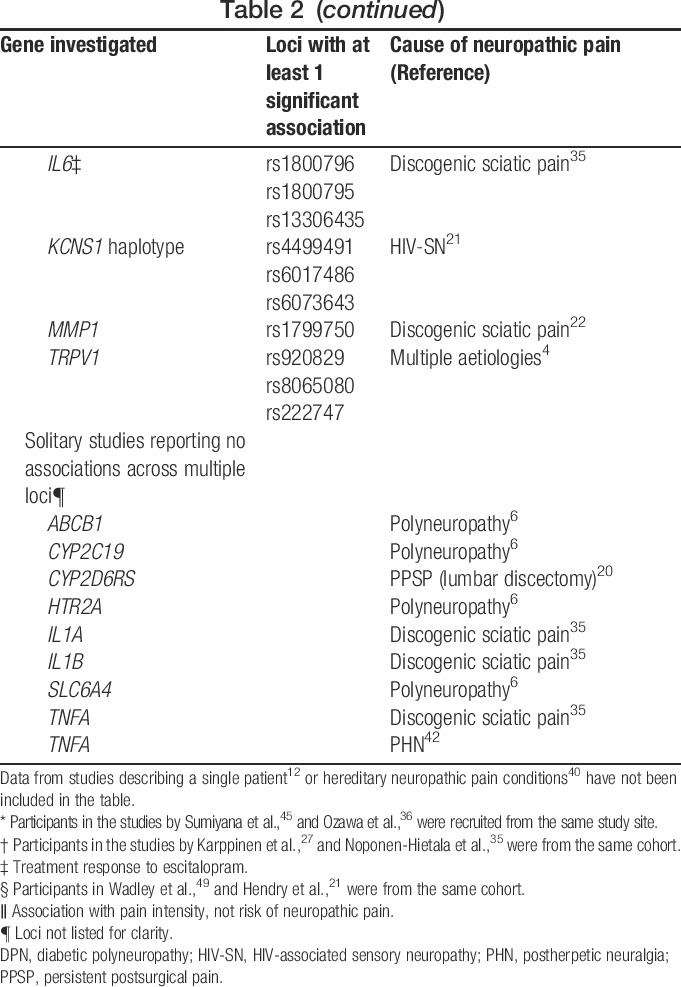
Systematic review: summary of genetic associations identified with neuropathic pain conditions.

#### 3.1.3. Phenotyping methods

The identified studies could be classified into 3 broad categories, according to their reason for phenotyping neuropathic pain: (1) to determine whether any pain was neuropathic (identification of cases and controls)^2,4,8,12,15,16,20,22,23,34–36,42,45,49^; (2) to identify endophenotypes within cohorts with neuropathic pain (ie, phenomics)^6,11,21,27,40,46,49^; and (3) to identify pain (not specifically neuropathic) in a neurological condition, eg, multiple sclerosis.^16^

Table 3 and Supplementary Digital Content 2 (available online as Supplemental Digital Content at http://links.lww.com/PAIN/A148) provide summaries of the phenotyping methods described in these articles. Overall, “clinical examination” was the most frequently reported phenotyping method; described in 15/21 studies, with “brief examination” reported in 6 studies, including in total 1742 cases, and “detailed examination” reported in 9 studies (1346 cases). “Pain-rating scales” (either visual analogue or numerical rating scales, 2786 cases) and “history” (2552 cases) were the next most common phenotyping method described, with 13/21 studies each. With “Pain-rating scales,” only half of these articles described what the relevance of the pain score was: for example, to be used as a cutoff for “caseness” to categorize patients as “cases” or “controls.” Nine of the articles reporting a “history” provided a “brief history” (2226 cases), and 4 of the articles provided a “detailed history” (326 cases). All other phenotyping methods were described in fewer than half of the articles, with the least frequently reported phenotyping methods being “nerve conduction studies” (2/21), “intraepidermal nerve fibre density” (2/21), “inflammatory markers” (2/21), “body chart of perceived pain” (1/20), and related “psychiatric measures” (1/21). In 2 studies, there was phenotyping heterogeneity, manifesting in the use of more than 1 sample population cohort within the same study.^11,15^

**Table 3:**
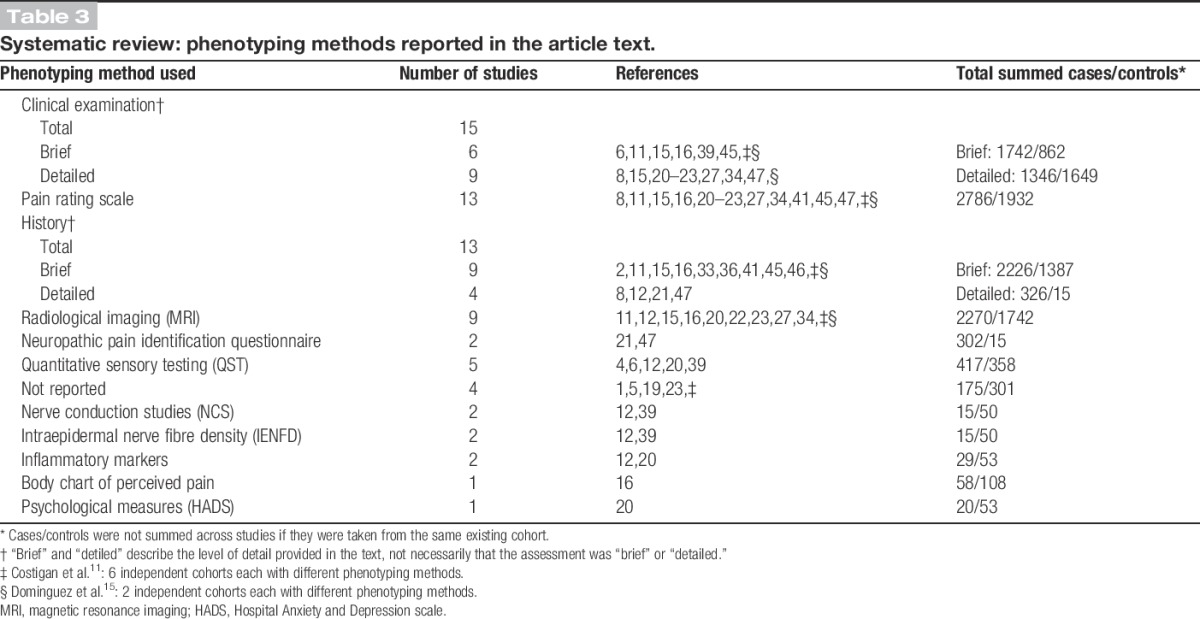
Systematic review: phenotyping methods reported in the article text.

The only specific neuropathic pain symptom questionnaire reported was the AIDS Clinical Trials Group Brief Peripheral Neuropathy Screen.^9,21,49^ No studies reported the use of validated case ascertainment tools such as the Douleur Neuropathique en 4 questions (DN4),^5^ Leeds Assessment of Neuropathic Symptoms and Signs (LANSS),^3^ or painDETECT.^18^

The control groups described in the studies could be divided into 3 broad categories: (1) healthy volunteers,^2,4,16,35,36,42,45^ (2) population-specific national reference cohorts,^15,22,23^ and (3) diseased controls.^6,8,15,16,20,27,34,49^ Three studies used more than 1 category of control group.^15,16,45^ In the first 2 categories (healthy controls and population-specific national reference cohorts), little or no information was provided on the phenotyping of controls. Typically, the information provided in these studies was restricted to whether the controls were healthy, genetically unrelated, and matched the cases for various demographic variables. Diseased controls were defined as having the same disease or aetiology as the cases, but were not considered to have neuropathic pain at the time of the study.

Four studies modelled genotype against intensity of neuropathic pain and thus did not include control groups.^11,21,46,49^ Similarly, 2 case reports did not include control groups at all.^12,40^

#### 3.1.4. Grading framework: “possible,” “probable,” or “definite” neuropathic pain

Only 1 of the 20 studies cited the NeuPSIG neuropathic pain grading system, and that single study indicated that cases satisfied the criteria for “definite” neuropathic pain.^40^ Table 4 summarises the grading criteria met by each study based on the description of the phenotyping methodology in the article text. Only 1 study did not describe assessing a history.^6^ Although only 1 study reported using a body chart of perceived pain^16^ to assess whether pain occurred in a plausible anatomical distribution, the assessment of pain distribution could be inferred from the phenotyping descriptions provided in 13 studies, even if the method of assessment was not explicitly described.^8,11,12,15,20–23,27,34,35,49^ Fourteen studies described assessing clinical signs,^6,8,12,15,16,20–23,27,35,40,49^ and 8 studies described the use of methods that could identify a nerve lesion (either radiological imaging and/or QST).^4,6,12,20,22,23,27,35^ In summary, 4 studies included patients identified as “possible” neuropathic pain cases,^11,34,45,46^ 5 included patients identified as “probable” neuropathic pain cases,^8,15,16,21,49^ and only 7 included patients identified as “definite” neuropathic pain cases.^12,20,22,23,27,35,40^ Six studies could not be graded according to the criteria proposed by Treede and et al.,^47^ and were classified as “undefined.”

**Table 4:**
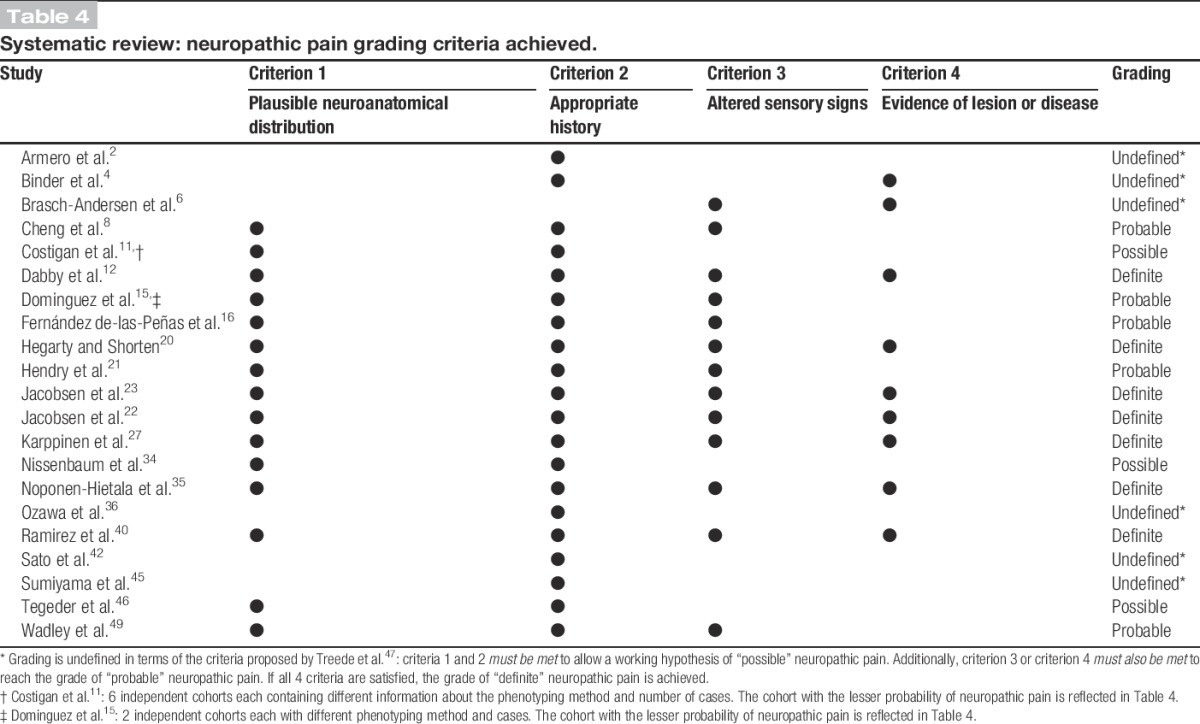
Systematic review: neuropathic pain grading criteria achieved.

### 3.2. Delphi survey

#### 3.2.1. Participation

Twenty experts, of the 28 approached to take part in the survey, completed round 1 (responder rate: 20/28, 71%), and 17/20 (85%) indicated that they were willing to participate in subsequent survey rounds. Round 2 invitations were sent to 17 experts, 16 of whom completed the questionnaire (responder rate: 16/17, 94%). Invitations to round 3 of the survey were sent to the same round 2 17 experts, 15 of whom completed the questionnaire (responder rate: 15/17, 88%).

#### 3.2.2. Validity and feasibility of assessing symptoms, signs, and completing additional investigations

**Figure 2:**
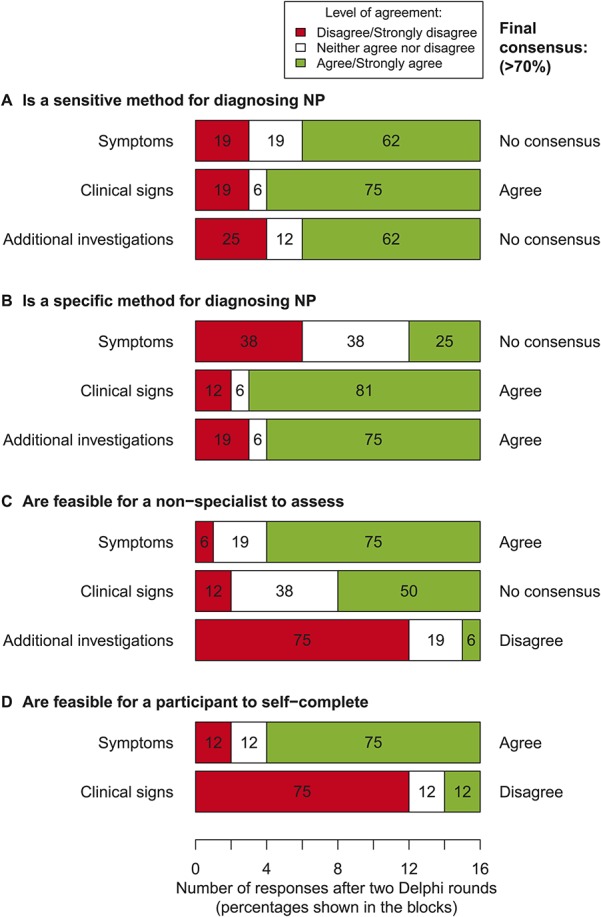
Delphi survey: level of agreement (5-point Likert scale, anchored at 1 = strongly disagree and 5 = strongly agree), and whether consensus was achieved (when ≥70% of respondents disagreed/strongly disagreed, or vice versa, agreed/strongly agreed with a statement) after 2 rounds of the survey (n = 16 in the second round). (A) Agreement on whether symptoms, clinical signs, and additional investigations are sensitive methods of detecting neuropathic pain. (B) Agreement on whether the 3 measurement domains are specific methods for detecting neuropathic pain. (C) Agreement on whether it is feasible for a nonspecialist to assess each of the 3 measurement domains in a research setting. (D) Agreement on whether it is feasible for study participants to self-assess symptoms and clinical signs.

Figure 2 shows the level of agreement of respondents after 2 survey rounds with regards to whether symptoms, clinical signs, and additional investigations are sensitive and specific assessments of neuropathic pain, and whether they are feasible for nonexperts and study participants to complete by themselves. There was good consensus that testing clinical signs was a sensitive method of detecting neuropathic pain as means of identifying true “cases,” but poor consensus was achieved for either symptoms or additional investigations alone. There was good consensus that clinical signs and additional investigations (but not symptoms) were specific methods to enable the identification of true “controls.” There was good consensus that symptoms could be reliably assessed by nonexperts and by study participants, but that additional investigations were not feasible for nonexperts or study participants to assess, and that study participants could not reliably self-assess clinical signs. The levels of agreement across each round of the survey are shown in Supplementary Digital Content 3 (available online as Supplemental Digital Content at http://links.lww.com/PAIN/A149).

#### 3.2.3. Informative symptoms, signs, and additional investigations

Table 5 shows the final consensus on respondents' choices and rankings of symptoms used in the diagnosis of neuropathic pain for a genetic study. Strong or good consensus was achieved for only 2 symptoms: “hot/burning” and “pain evoked by light touch.” Fewer than one-third of respondents included “spontaneous pain,” “paroxysmal pain,” “evoked pain,” “painful cold,” “itching,” or “stabbing pain” in their top 5 symptoms for diagnosing neuropathic pain.

**Table 5:**
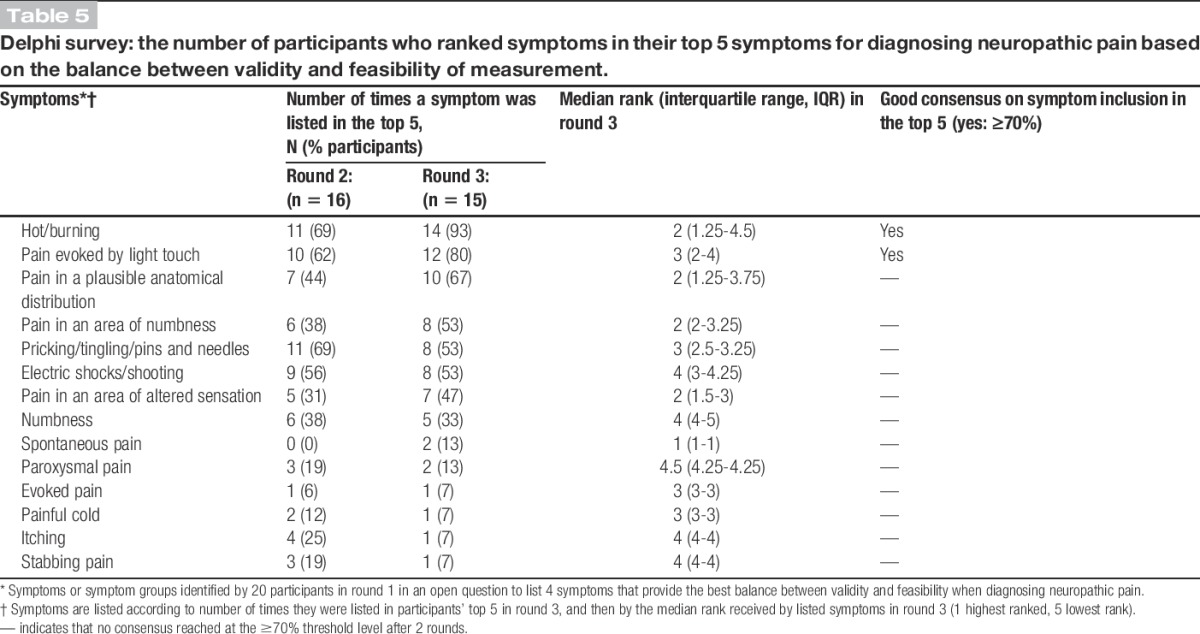
Delphi survey: the number of participants who ranked symptoms in their top 5 symptoms for diagnosing neuropathic pain based on the balance between validity and feasibility of measurement.

Respondents' choices and rankings of clinical signs used in the diagnosis of neuropathic pain are shown in Table 6. Strong or good consensus was achieved for only 2 signs: “dynamic mechanical allodynia” and “altered sensation to punctate mechanical stimuli.” Fewer than one-third of respondents included “temporal summation,” “altered vibration sense,” “thermal hyperalgesia,” “altered reflexes,” “static mechanical hyperalgesia,” or “deep mechanical hyperalgesia” in their top 5 signs to assess.

**Table 6:**
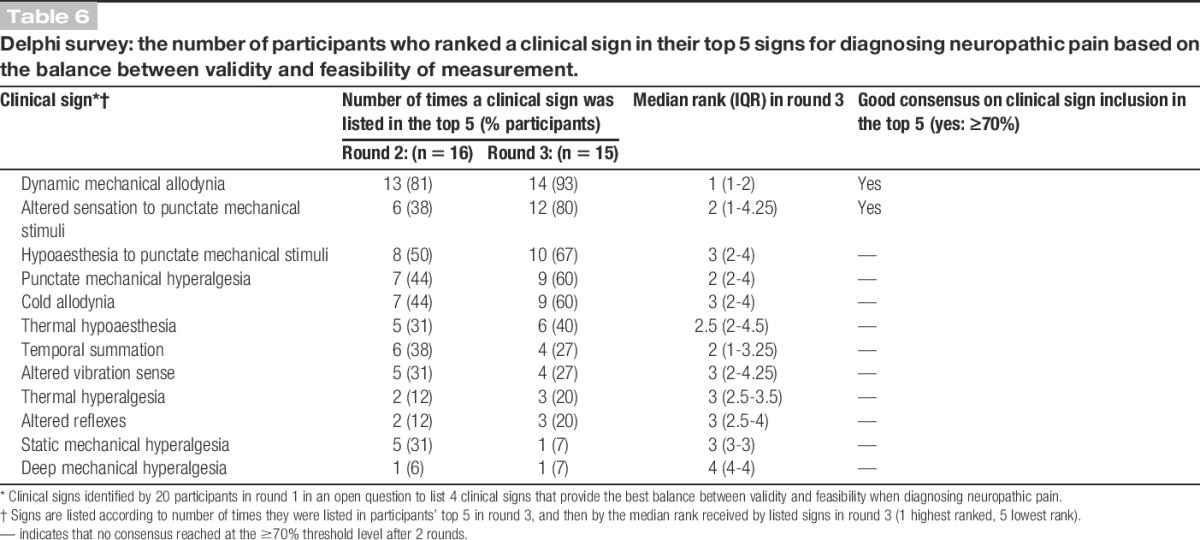
Delphi survey: the number of participants who ranked a clinical sign in their top 5 signs for diagnosing neuropathic pain based on the balance between validity and feasibility of measurement.

Table 7 shows the final consensus of respondents' choices and rankings of their top 5 additional investigations for diagnosing neuropathic pain. The 2 top-ranked investigations were, “QST” and “intraepidermal nerve fibre density.” In round 3, when the feasibility of nonspecialists making accurate measurements was assessed for each of the 5 additional investigations, only QST was rated as being feasible by 11/14 (79%) of respondents. More than 70% of respondents agreed that all other methods of assessment were not feasible for nonspecialists to assess neuropathic pain.

**Table 7:**
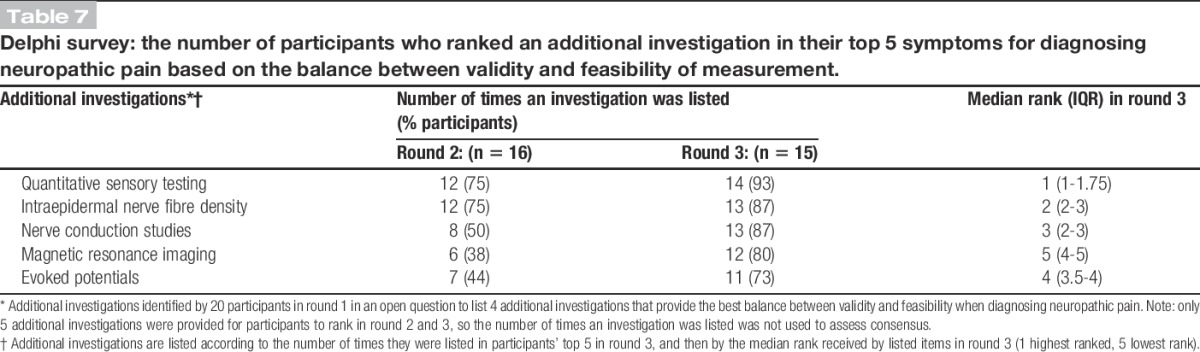
Delphi survey: the number of participants who ranked an additional investigation in their top 5 symptoms for diagnosing neuropathic pain based on the balance between validity and feasibility of measurement.

#### 3.2.4. Perceived diagnostic certainty

**Figure 3:**
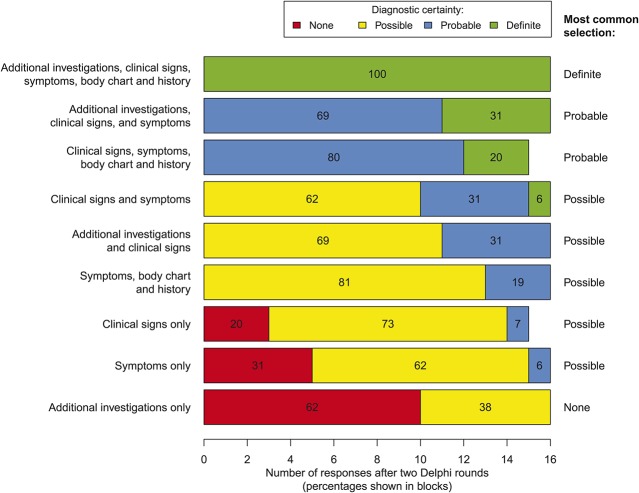
Delphi survey: level of diagnostic certainty achieved by individual assessment types and combinations of these assessments when assessing whether a pain is “definite,” “probable,” or “possible” neuropathic pain.^2^ Results shown are after 2 rounds of the Delphi survey (n = 16 in the second round).

Figure 3 shows the level of perceived diagnostic certainty, according to the neuropathic pain grading system,^47^ indicated by respondents for various assessment modalities used alone or in combination with each other.^2^ There was unanimous consensus that a combination of “additional investigations, clinical signs, symptoms, body chart, and patient history” provided a diagnosis of “definite” neuropathic pain. All respondents also indicated that combinations of: “additional investigations, clinical signs and symptoms,” and “clinical signs, symptoms, body chart, and patient history” provided a diagnosis of at least “probable” neuropathic pain. The poorest perceived diagnostic certainty was associated with assessments that only included “additional investigations.” The levels of agreement across each round of the survey are shown in Supplementary Digital Content 4 (available online as Supplemental Digital Content at http://links.lww.com/PAIN/A150).

#### 3.2.5. Additional phenotyping measures

The level of agreement on which measurements should be included in an assessment of neuropathic pain (in addition to symptoms, signs, and additional investigations) is shown in Table 5 (and Supplementary Digital Content 5, available online as Supplemental Digital Content at http://links.lww.com/PAIN/A151). After 2 rounds of the survey, there was strong consensus that assessment should include a history and a body chart to indicate pain distribution. Prompted by discussions at the consensus meeting (see below), in round 3 of the survey, we probed what the body chart and history should include. Having ≥70% of respondents in agreed or strong agreement, the strongest consensus was for the following items assessing patient history: “a previous diagnosis of neuropathic pain by another clinician” and “risk factors for having neuropathic pain” (see Supplementary Digital Content 6, available online as Supplemental Digital Content at http://links.lww.com/PAIN/A152). On the body chart of perceived pain, 75% (12/16) of respondents indicated that, in the event of more than 1 pain site, the “main pain *and* any other pains” should be identified. Almost two-thirds (64%, 9/14) believed that a checklist of body regions was a suitable substitute for a body chart cartoon.

### 3.3. Consensus meeting

The primary goal of the meeting was the development of a consensus statement on “entry level” phenotyping requirements for genetic studies of neuropathic pain in human adults.

#### 3.3.1. Consensus statement development

Participants agreed that there is an inverse relationship between accurate case ascertainment of neuropathic pain and large-scale implementation thereof in population-based studies. We agreed that the entry-level phenotyping requirements included in the statement should form the basis of establishing neuropathic pain “caseness” for genetic studies. This basic phenotyping must allow the addition of more in-depth measures for higher level phenotyping to allow the identification of genetic loci for specific aspects of neuropathic pain other than just presence or absence of neuropathic pain. The entry-level requirements should provide a framework to guide researchers on study design and provide a platform to appraise their findings to allow feasible and valid assessment for the presence or absence of neuropathic pain in large population-based genetic studies.

#### 3.3.2. “Entry level” phenotyping for genetic studies, a consensus

Box 1 shows the agreed NeuroPPIC entry-level requirements that would allow the classification of participants as having “possible” neuropathic pain. Consistent with the findings of the Delphi survey, a neuropathic pain “case” must have:

(1) pain with neuropathic characteristics (ie, positive on a validated screening questionnaire; or pain, ie, “hot/burning” and/or “pain evoked by light touch”);
(2) a distribution or location of this pain that is anatomically consistent with an underlying somatosensory lesion or disease (as indicated by a body chart or body parts checklist); and
(3) further characterisation in the form of a clinical history (duration and intensity of pain and presence of other pains) and demographic information relevant to the population/disease being studied.

Box 1 **NeuroPPIC consensus statement on entry-level phenotyping for genetic studies of neuropathic pain in humans.**

**Table:**
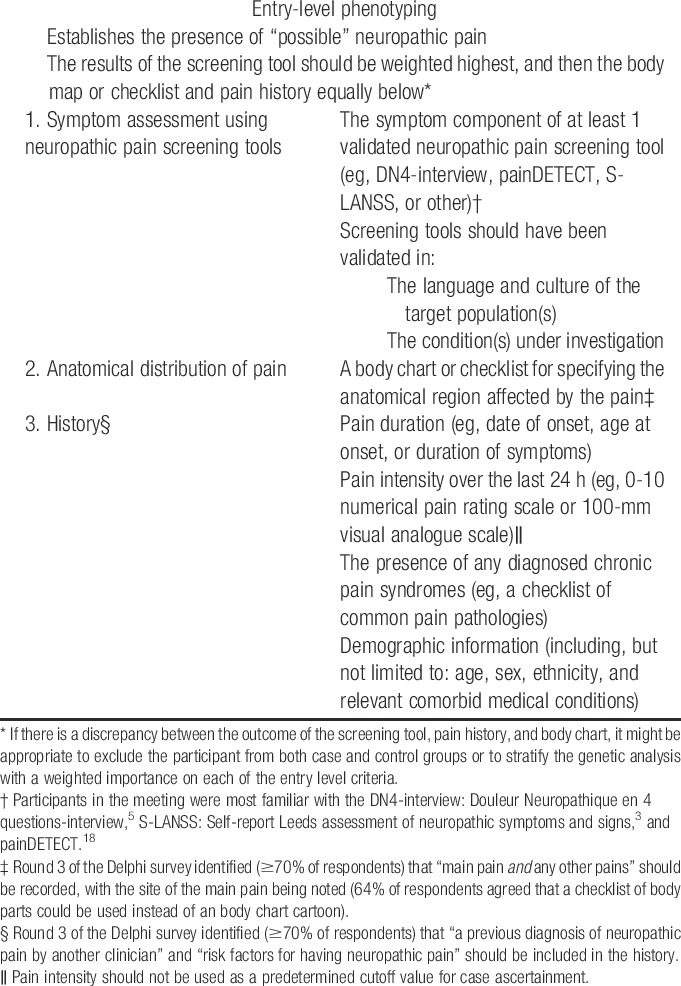

A neuropathic pain “control” would be negative for (1) and (2), but will be characterised as (3) as clinically relevant.

The panel agreed that if there are discrepancies between the findings from the screening tool, anatomical distribution, and patient history, then the participant should be excluded from the study. Overall, these 3 components only satisfy criteria 1 and 2 of the NeuPSIG grading system, and thus the level of diagnostic certainty achieved is “possible” neuropathic pain.^47^

While acknowledging the lack of a “gold standard,” we agreed that achieving greater diagnostic certainty (ie, “probable” or “definite” neuropathic pain) is desirable. Such greater diagnostic certainty requires additional assessments that confirm the presence of positive and/or negative sensory signs (for “probable” neuropathic pain) or provide direct evidence of a lesion to the somatosensory nervous system (for “definite” neuropathic pain) in the affected area. More extensive phenotyping could allow case stratification into additional pain phenotypes based on sensory and psychological parameters (eg, pain catastrophizers vs noncatastrophizers; pinprick hypoaesthesia vs pinprick hyperaesthesia).

The panel additionally agreed that it could not currently be prescriptive in the selection of additional phenotyping measures to use beyond determination of “caseness,” as the choice was dependent on factors such as the feasibility of making measurements based on cost and practicality, the population and disease being studied (eg, central vs peripheral neuropathic pain), and the research question being addressed. However, choice of additional measures to use when assessing “caseness” should be consistent with the NeuPSIG grading system for neuropathic pain. See Supplementary Digital Content 7 for examples of additional phenotypes voiced at the meeting (available online as Supplemental Digital Content at http://links.lww.com/PAIN/A153).

#### 3.3.3. Misclassification

We agreed that misclassification of cases and controls significantly affects the power of studies to detect true genetic associations and the reproducibility of findings. We identified several key factors that may contribute to misclassification of cases and controls (Box 2). These factors can be divided into 2 broad categories: (1) misclassification due to on incomplete pain history; and (2) misclassification due to an imperfect sensitivity and specificity of available assessments for neuropathic pain. Ideally, where reasonable doubt arises after applying the phenotyping criteria proposed here, an individual should be considered as neither a case nor a control, and removed from analysis.

Box 2 **Possible factors that may contribute to misclassification of neuropathic pain.**

**Table:**
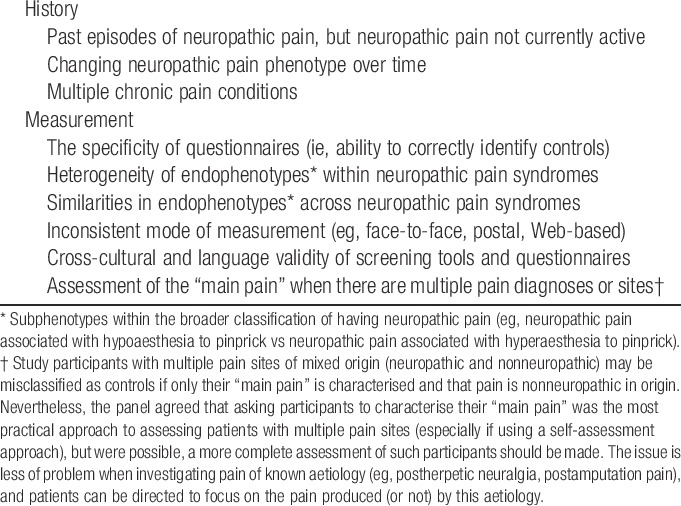

## 4. Discussion

The unequivocal identification of genetic sequence variants correlating with complex diseases requires sample sizes ranging from large cohorts (including hundreds to thousands of participants) when interrogating a few candidate genes, to much larger sample sizes that include tens of thousands of participants when studying the whole genome^31,51^ and extensive phenotyping.^50^ Unlike Mendelian disorders, the genetic contribution to complex diseases such as chronic pain is generally attributed to contributions of a high number of risk variants, including single-nucleotide polymorphisms, most with a small effect. In neuropathic pain, a number of relevant candidate genes have been identified in animal studies, and an even smaller number show some association in humans, as shown above. However, none of these single-nucleotide polymorphisms has been consistently replicated in large human cohorts.^32^ This is an essential step in confirming the validity of these genetic findings.^7^ Failure to replicate, in humans, a finding originally made in humans may be because the original finding was spurious, study cohorts were underpowered, there was heterogeneity in the studied pain condition or study populations, or there were differences in the phenotyping of cases and/or controls. The ideal control in a genetic study would be an age- and sex-matched individual who was exposed to the same lesion or disease of the somatosensory system as the case, but who did not develop neuropathic pain.^30^ Rigorous, standardised phenotyping is therefore crucial. Failure to translate a finding from rodent models to humans may likewise be because the original finding was spurious, the human cohort was underpowered, the phenotypes were different, or the gene under study does not play a role in the tested human condition. Pain geneticists who seek candidate neuropathic pain genes in animal models should try to approximate their phenotypes to those used by human pain geneticists, although animal phenotypes also require considerable further refinement in terms of both internal and external validity.^37^

The need for a consensus-based, rather than purely evidence-based approach to phenotyping neuropathic pain for genetic studies reflects the absence of “gold standards” for both case definition (what *is* neuropathic pain?) and case attribution (how do we know who *has* neuropathic pain?). Through a process of systematic literature review, Delphi survey, and expert consensus meeting, we have identified an agreed “entry level” approach to phenotyping cases and controls for studies of genetic associations with neuropathic pain in adults. This phenotype is based on establishing the presence or absence of “possible” neuropathic pain, according to the most internationally agreed system for classifying neuropathic pain clinically.^47^ This uses validated symptom-based screening tools and a body chart or checklist to determine neuroanatomical relevance. Further details, also collected by questionnaire, include pain history (duration, intensity, and formal diagnoses) and demographic information (Box 1). We present this “entry level” approach as the minimum phenotyping standard for genetic association studies on neuropathic pain in adults, with the expectation that more extensive phenotyping should build on this “entry level” approach, as required and as resources allow. Adoption of these NeuroPPIC phenotyping guidelines will facilitate comparison and collaboration between researchers, including replication studies and meta-analyses. The guidelines are applicable to different types of genetic studies of neuropathic pain, from targeted studies interrogating a few genetic loci that use specific neuropathic pain traits to hypothesis-free genome-wide studies with large sample sizes. They will also apply to studies with a larger array of phenotypes, including more detailed pain traits, psychosocial traits, and pharmacological screens. Our systematic review identified the lack of a consistent approach to phenotyping neuropathic pain, poor reporting of phenotyping methodologies, and failure to adequately describe control groups (especially when “healthy controls” were used). These limitations are likely to have contributed to inconsistent and nonreplicated findings, and highlight the need for the NeuroPPIC process we have completed.

Although we have recommended the use of validated assessment instruments where possible, our guidelines are not intended to guide case definition, attribution, or classification for clinical research or neurological practice, where more individuals certainty may be required, based on empirical evidence of the presence or absence of neuropathic pain and the known likelihood of identifying this through any assessment. This will require separate work, and is the subject of an ongoing NeuPSIG project. Instead, our consensus-based development provides a standardised approach for use by researchers in different settings internationally, in the expectation that it will allow a common approach to identifying the presence or absence of “possible” neuropathic pain for genetic research. However, future research should validate the “entry level” phenotype against best available clinical assessments, with a view to calculating sensitivity, specificity, and positive and negative predictive values.

Delphi surveys are an accepted method of achieving consensus on complex issues in science and medicine,^13,39^ including pain medicine, in which a “gold standard” does not exist (as in neuropathic pain^26^). For example, previous studies have used Delphi methods to develop a standard definition for back pain^14^ and neuropathic pain^44^ for use in prevalence studies, and to develop guidelines for the management of hip and knee osteoarthritis.^52^ Identified limitations of the method include its basis in the opinions of a selected group of survey participants, and the potential for the outcome to be a diluted version of “best opinion,”^39^ criticisms that could also be levelled at consensus meetings. There is no agreed optimum number or composition of panellists or rounds for Delphi surveys or consensus meetings. Our intention was to include a range of disciplines rather than to be comprehensive in our inclusion, and to include a similar number of participants and rounds as in previous successful studies.^32,44,52^ This would allow the processes to remain manageable, while ensuring a contribution of expertise from each relevant discipline and consistency with existing research. Contributors were identified on the basis of their published track record in neuropathic pain phenotyping and/or genetics. It is possible that a different group of contributors would have produced different outcomes, and unanimity was not obtained for any item in the survey. It is, though, notable that good or very good consensus was achieved in many useful aspects of this Delphi survey, and agreement on the NeuroPPIC “entry level” phenotyping approach was achieved after intense deliberations. We are, therefore, confident that our findings reflect the views of the relevant research community, resulting in adoption of NeuroPPIC's approach in future genetic studies.

NeuroPPIC has only provided a consensual statement on the nature of “entry level” phenotyping and instruments that can be used to accomplish successful classification of participants as having vs not having neuropathic pain. These NeuroPPIC guidelines are for the basic phenotyping requirements for any genetic study of neuropathic pain in adults. It is notable that this “entry level” phenotyping is based only on study participants' responses to questions and does not include clinical examination or further investigations. This “entry level” phenotyping approach will allow inclusion in large-scale questionnaire-based surveys. Our Delphi survey confirms that augmenting a simple symptom checklist by questions on pain bodily distribution and history will allow the identification of “possible” neuropathic pain. Although symptoms could be confined to a smaller list of verbal descriptors (eg, “hot/burning pain” and “pain evoked by light touch”), the use of a slightly longer but well-validated screening instrument will prevent the need for validation of a new questionnaire instrument. Available screening instruments include DN4, S-LANSS, and PainDETECT, but we are making no statement on which of these, if any, is to be preferred. This approach conforms to the “very general” approach outlined in the introduction and will be suitable for large-scale population-based studies, including those in which neuropathic pain is just one of a number of conditions to be phenotyped. More extensive phenotyping of additional aspects of neuropathic pain will require a more detailed set of assessments, to allow for inclusion of “very specific” phenotypes. Although NeuroPPIC was able to provide some pointers towards these levels, we have not yet furnished agreed guidelines on the nature of these phenotypes and the instruments to collect them, and this will require further work, building on the “entry level” approach. Any additional, more extensive phenotyping should, however, be consistent with the “entry level” approach, to allow specificity to be built in to the basic model. This will allow incremental approaches to phenotyping within a large cohort, and the testing for replication of associations identified in extensively phenotyped cohorts.

### 4.1. Reporting recommendations

The STREGA^29^ and GRIPS^24,25^ guidelines provide a framework for reporting genetic epidemiology studies and indicate that participant eligibility criteria and methods of participant selection and screening, including subsets of participants, should be reported. Moreover, in case–control studies, this information should be reported for both cases and controls. To facilitate clear and open reporting of phenotyping in genetic studies of neuropathic pain, we propose the following NeuroPPIC guidelines for describing study participants: (1) a description of sampling methodology and participant eligibility criteria; (2) an unambiguous description of the case definition used to define the presence or absence of neuropathic pain in cases and controls; (3) a description of the methods used to assess each component of the case definition and the criteria used to define abnormal function for each method used; and (4) a summative statement of whether the case definition satisfies the criteria for “possible,” “probable,” or “definite” neuropathic pain based on the latest grading guidelines (currently Treede et al.^47^). These recommendations are consistent with the recommendations made by STREGA and GRIPS and should be used in addition to those 2 guidelines.

By providing an “entry level” phenotype and making recommendations on the reporting of the criteria and methods used for caseness and its ascertainment, we believe that greater consistency and transparency can be achieved in studies on the genetics of neuropathic pain in adult humans. These improvements will facilitate advancements in the field by enabling collaboration between research groups, replication of discoveries of contributing genetic variants, meta-analyses, and translation from the laboratory to the general population, and back again.

## Conflict of interest statement

P. R. Kamerman, N. Attal, M. Haanpää, S. N. Raja, A. S.C. Rice, and B. H. Smith were members of the NeuPSIG Management Committee at the time of the research. O. van Hecke, D. L.H. Bennett, L. Diatchenko, R. Freeman, M. Haanpää, Z. Seltzer, and D. Yarnitsky declared no other conflicts of interest. N. Attal declared consultancy or lecture fees from Pfizer, AstraZeneca, Lilly, Grunenthal, Johnson and Johnson Sanofi Pasteur Mérieux outside the scope of this work. P. R. Kamerman declared consultancy fees from Reckitt Benckiser, lecture fees from Pfizer and Novartis, and travel support from Janssen. B. H. Smith declared occasional consultancy and lecture fees, on behalf of his institution, from Pfizer, Napp, and Grunenthal. He is the Scottish Government Lead Clinician for Chronic Pain. R. Baron has received grants/research support from Pfizer, Genzyme, Grünenthal, and Mundipharma. He is a member of the IMI “Europain” collaboration and industry members of this are: AstraZeneca, Pfizer, Esteve, UCB-Pharma, Sanofi-Aventis, Grünenthal, Eli Lilly, and Boehringer Ingelheim. German Federal Ministry of Education and Research (BMBF): German Research Network on Neuropathic Pain, NoPain system biology. German Research Foundation (DFG). He has received speaking fees from Pfizer, Genzyme, Grünenthal, Mundipharma, Sanofi Pasteur, Medtronic, Eisai, Lilly, Boehringer Ingelheim, Astellas, Desitin, Teva Pharma, Bayer-Schering, and MSD. He has been a consultant for Pfizer, Genzyme, Grünenthal, Mundipharma, Allergan, Sanofi Pasteur, Medtronic, Eisai, Lilly, Boehringer Ingelheim, Astellas, Novartis, Bristol-Myers-Squibb, Biogenidec, AstraZeneca, Merck, AbbVie, Daiichi Sankyo, and Glenmark Pharmaceuticals. G. Bjornsdottir and T. E. Thorgeirsson are employed by deCODE Genetics/Amgen Inc and have received funding from the European Commission (HEALTH-FP7-2013-Innovation-602891 to the NeuroPain Consortium) and the NIH (R01DE022905). D. L.H. Bennett declared occasional consultancy and lecture fees, on behalf of his institution, from Pfizer. M. I. Bennett declared consultancy and lecture fees from Pfizer, Bayer, and Grunenthal. R. Freynhagen declared research support, consulting, or lecture fees in the past 2 years from Astellas, Grünenthal, Janssen, Lilly, and Pfizer. T. S. Jensen declared consultancy fees for Pfizer, Grünenthal, Orion, and Astellas and lecture fees from Pfizer. S. N. Raja declared a research grant from Medtronic and that he has served as an Advisor for Mitsubishi Tanabe Pharma Inc. A. S.C. Rice declared Imperial College Consultants Consultancy work and Scientific Board Memberships contracted by Imperial College Consultants. In the last 3 years, these have included remunerated work for the following companies: Spinifex, Merck, Astellas, Medivir, Asahi Kasei Pharma, Servier, Relmada, Abide, Neusentis (Pfizer), Aquilas (Neuromax), and Mitsubishi; and ownership of share options in Spinifex. He also declared receipt of research grant support in the last 3 years, on behalf of his institution, from Pfizer (as part of London Pain Consortium and Neuropain) and Astellas (as part of EU Innovative Medicines Initiative grant-EUROPAIN). He is a member of the UK Department of Health (nonfinancial) Joint Committee on Vaccination and Immunisation, Varicella/Herpes Zoster subgroup. M. Haanpää declared consultancy fees for AbbVie, Allergan, Astellas, Eli Lilly, Janssen Cilag, Pfizer, and Sanofi-Aventis, and lecture fees from Astellas, Eli Lilly, Janssen Cilag, MSD, Mundipharma, Orion, and Pfizer. D. L.H. Bennett is a Wellcome Senior Clinical Scientist (Ref 095698z/11/z). N. Attal, D. Bouhassira, D.L.H. Bennett, R. Baron, T. S. Jensen, A. S.C. Rice, B. H. Smith, and D. Yarnitsky are members of the DOLORisk consortium funded by European Commission Horizon 2020 (ID633491).

This work was entirely funded by the Neuropathic Pain Special Interest Group (NeuPSIG) of the International Association for the Study of Pain.

O. van Hecke and P. R. Kamerman contributed equally to this work.

## Supplementary Material

SUPPLEMENTARY MATERIAL

## Appendices Supplemental Digital Content

Supplemental Digital Content associated with this article can be found online at http://links.lww.com/PAIN/A147; http://links.lww.com/PAIN/A148; http://links.lww.com/PAIN/A149; http://links.lww.com/PAIN/A150; http://links.lww.com/PAIN/A151; http://links.lww.com/PAIN/A152; http://links.lww.com/PAIN/A153.
