# Supplementary material for: Neuropathic pain phenotyping by international consensus (NeuroPPIC) for genetic studies: a NeuPSIG systematic review, Delphi survey, and expert panel recommendations
Source: Pain. 2015 Oct 22;156(11):2337–53. doi: 10.1097/j.pain.0000000000000335 (PMC4747983; doi:10.1097/j.pain.0000000000000335)
Supplement: SUPPLEMENTARY MATERIAL [file jop-156-2337-s001.pdf]

Supplementary Digital Content 1

Table. Sample search strategy (January 1966 to April 2014)

Search number	Term(s)
1.	polymorphism.af
2.	SNP.af
3.	single nucleotide polymorphism.af
4.	genetics.af
5.	genet*.af
6.	GWAS.af
7.	Genome-Wide Association Study.af
8.	genetic predisposition to disease.af
9.	candidate genes.af
10.	allele.af
11.	genotype.af
12.	genetic association studies.af
13.	1 or 2 or 3 or 4 or 5 or 6 or 7 or 8 or 9 or 10 or 11 or 12
14.	neuropath*pain.af
15.	neurogenic pain.af
16.	deafferentation pain.af
17.	(neuropath* adj5 pain).af
18.	(neurogenic adj5 pain).af
19.	(deafferentation adj5 pain).af
20.	neuralgia.af
21.	nerve pain.af
22.	(nerve dysfunction or nerve damage or nerve destruction or somatosensory dysfunction).af
23.	peripheral neuropath*.af
24.	peripheral nerve injur*.af
25.	central neuropath*.af
26.	central nerve injur*.af
27.	polyneuropathy*.af
28.	pain.af
29.	14 or 15 or 16 or 17 or 18 or 19 or 20 or 21 or 22 or 23 or 24 or 25 or 26 or 27
30.	13 and 28 and 39
