# Supplementary material for: Neuropathic pain phenotyping by international consensus (NeuroPPIC) for genetic studies: a NeuPSIG systematic review, Delphi survey, and expert panel recommendations
Source: Pain. 2015 Oct 22;156(11):2337–53. doi: 10.1097/j.pain.0000000000000335 (PMC4747983; doi:10.1097/j.pain.0000000000000335)
Supplement: SUPPLEMENTARY MATERIAL [file jop-156-2337-s002.pdf]

Supplementary Digital Content 2

Table. Systematic review: phenotyping methods reported in the article text[a]

	Reference																				
	[2]	[4]	[6]	[8]	[11][b]	[12]	[14][c]	[15]	[20]	[21]	[22]	[23]	[27]	[35]	[36]	[37]	[41]	[43]	[46]	[47]	[49]
**Clinical examination**[d]																					
• Brief description			●		○		○	●							●		●			●	
• Detailed description				●		●			●	●	●	●	●								●
**Pain rating scale**				●	○		○	●	●	●	●	●	●		●			●		●	●
**History**[d]																					
• Brief	●				○		○	●						●		●		●	●	●	
• Detailed				●		●				●											●
**Radiological imaging (MRI)**					○	●	○	●	●		●	●	●		●					●	
**Neuropathic pain identification questionnaire**						●	○		●		●	●									
**Quantitative sensory testing (QST)**		●	●			●			●								●				
**Nerve conduction studies (NCS)**						●											●				
**Intra-epidermal nerve fibre density (IENFD)**						●											●				
**Inflammatory markers**						●			●												
**Body chart**								●													
**Psychological measures (HADS)**									●												

[a] Open circles relate to studies reporting on multiple cohorts, with different phenotyping methods described for each cohort;
[b] Costigan et al., 2010 [11]: Six independent cohorts each with different phenotyping methods;
[c] Dominguez et al., 2013 [14]: Two independent cohorts each with different phenotyping methods;
[d] '*brief*' and '*detailed'* describe the level of detail provided in the text of the paper, not necessarily that the assessment was '*brief*' or '*detailed*';
MRI: Magnetic resonance imaging;
HADS: Hospital Anxiety and Depression Scale
