# Supplementary material for: Neuropathic pain phenotyping by international consensus (NeuroPPIC) for genetic studies: a NeuPSIG systematic review, Delphi survey, and expert panel recommendations
Source: Pain. 2015 Oct 22;156(11):2337–53. doi: 10.1097/j.pain.0000000000000335 (PMC4747983; doi:10.1097/j.pain.0000000000000335)
Supplement: SUPPLEMENTARY MATERIAL [file jop-156-2337-s003.pdf]

Supplementary Digital Content 3

Figure. Delphi survey: level of agreement on whether symptoms (page 2), clinical signs (page 3), and additional investigations (page 4) were: (1) sensitive methods of detecting neuropathic pain, (2) specific methods for detecting neuropathic pain, (3) feasible for a non-specialist to assess in a research setting, and (4) feasible for study participants to self-assess symptoms and clinical signs.

Symptoms

(NP: neuropathic pain)

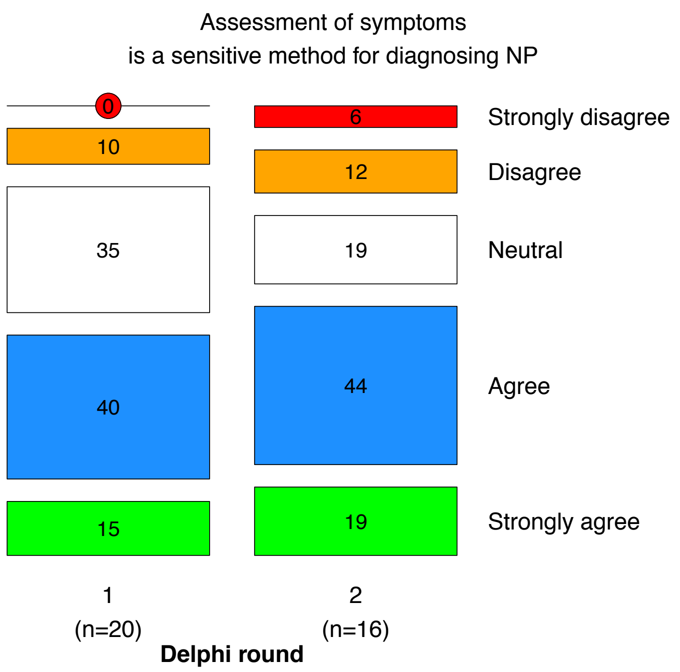

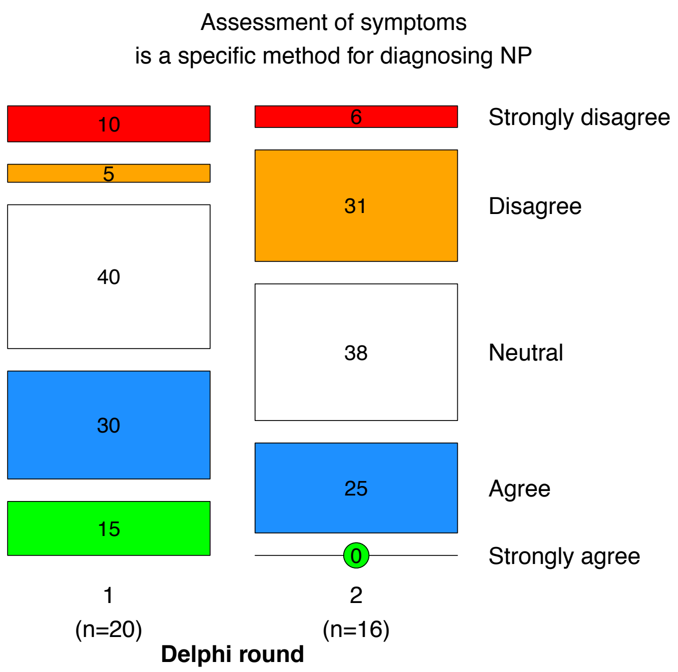

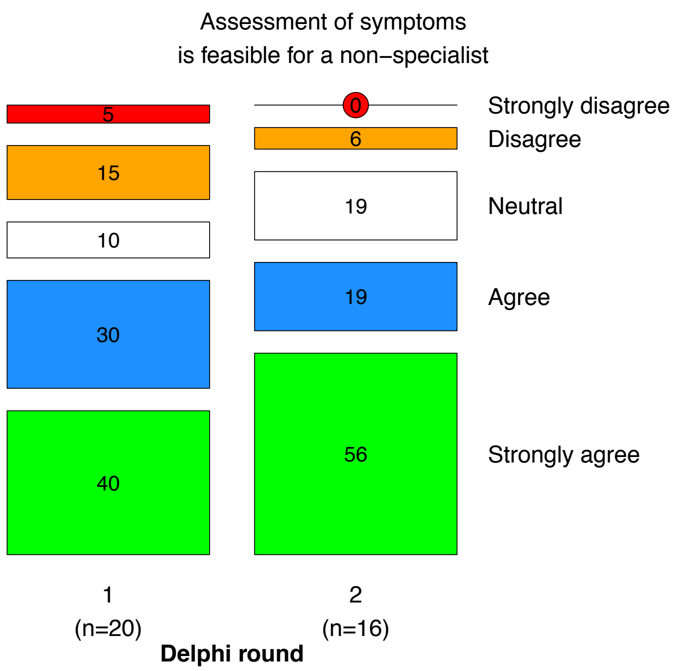

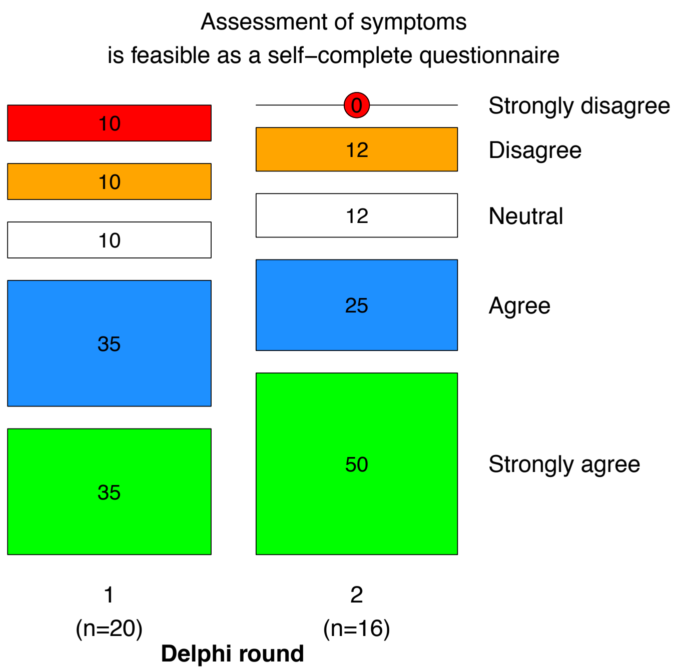

Numbers in the blocks report percent respondents choosing a category
Note: percentages may not match those presented in Table 1 because of rounding

Clinical signs

(NP: neuropathic pain)

Assessment of clinical signs
is a sensitive method for diagnosing NP

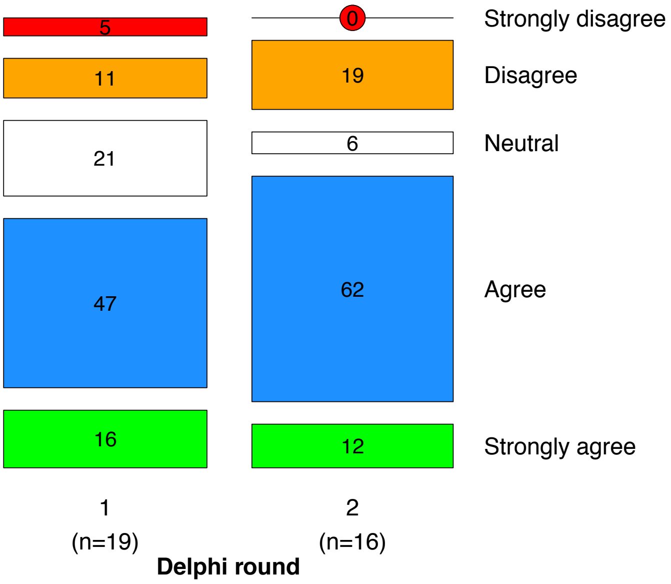

Assessment of clinical signs
is a specific method for diagnosing NP

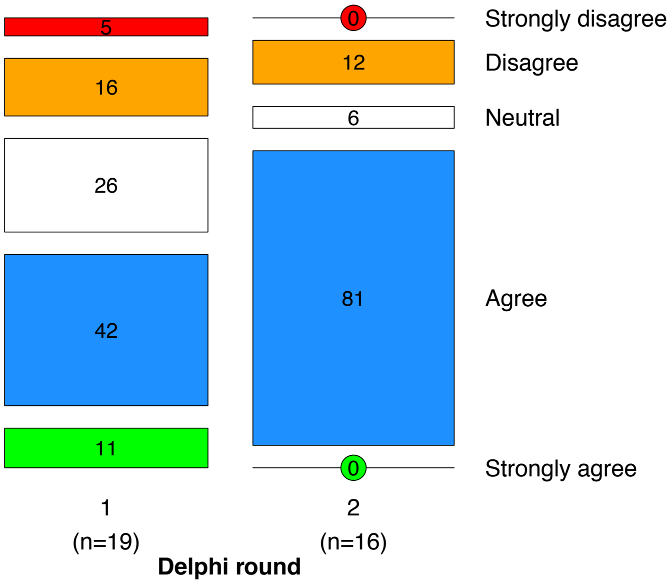

Assessment of clinical signs
is feasible for a non–specialist

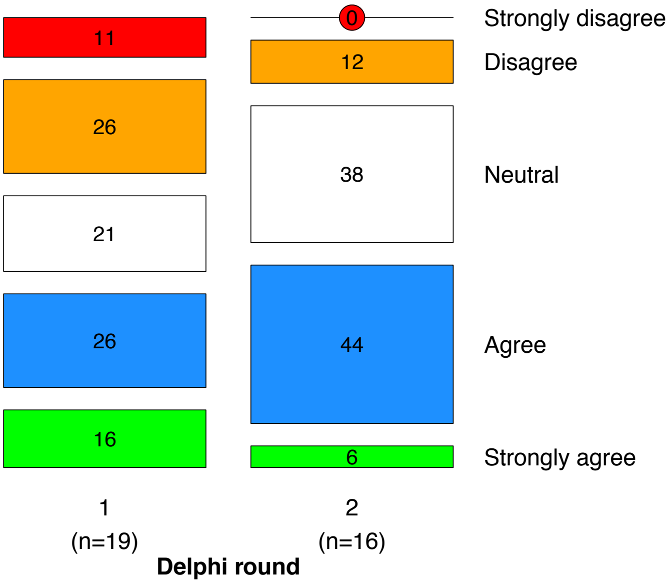

Assessment of clinical signs
is feasible by self–examination

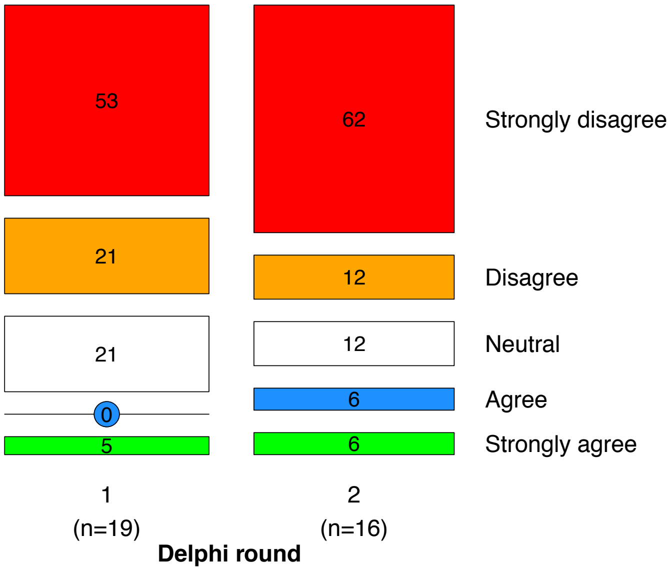

Numbers in the blocks report percent respondents choosing a category
Note: percentages may not match those presented in Table 1 because of rounding

Additional investigations

(NP: neuropathic pain)

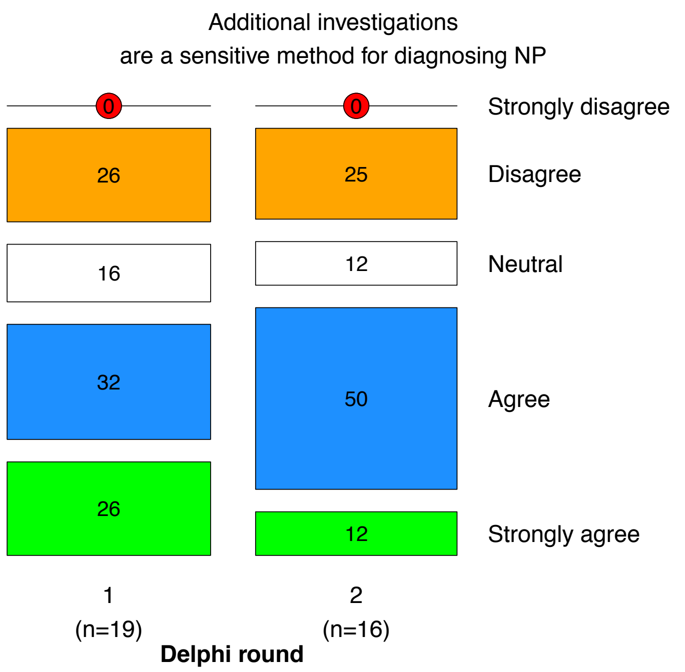

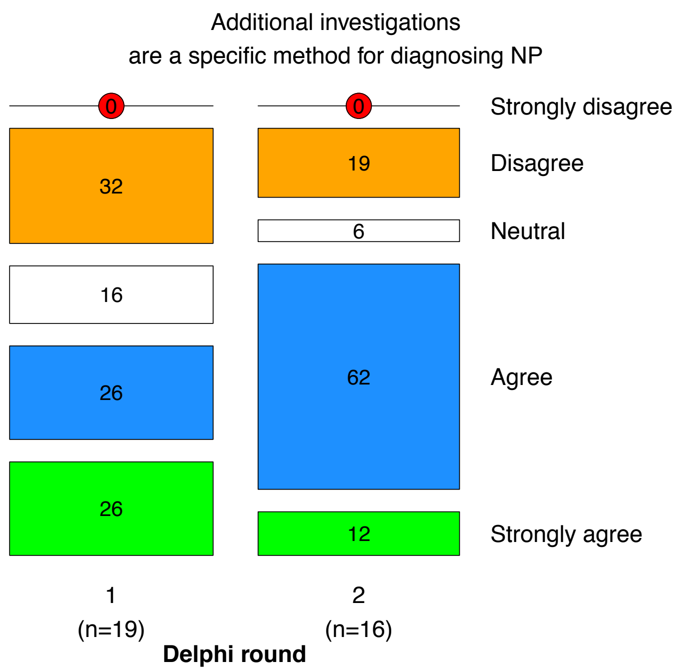

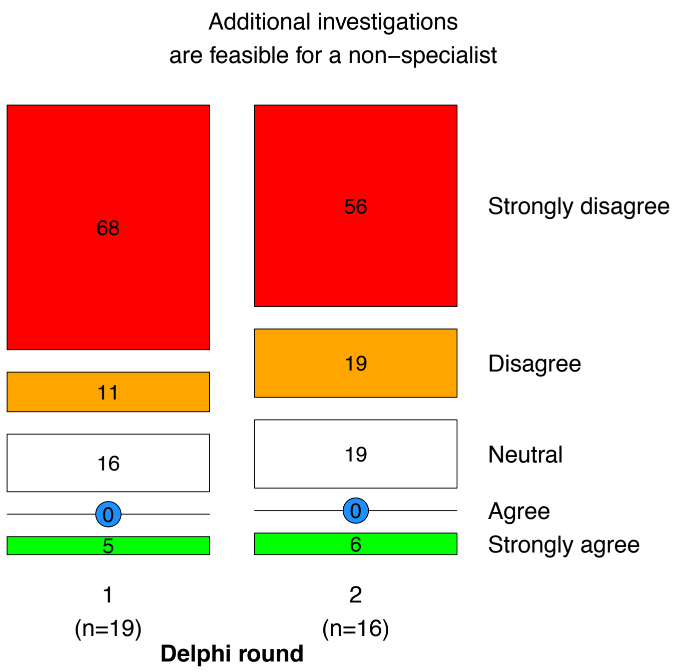

Numbers in the blocks report percent respondents choosing a category
Note: percentages may not match those presented in Table 1 because of rounding
