# Supplementary material for: Neuropathic pain phenotyping by international consensus (NeuroPPIC) for genetic studies: a NeuPSIG systematic review, Delphi survey, and expert panel recommendations
Source: Pain. 2015 Oct 22;156(11):2337–53. doi: 10.1097/j.pain.0000000000000335 (PMC4747983; doi:10.1097/j.pain.0000000000000335)
Supplement: SUPPLEMENTARY MATERIAL [file jop-156-2337-s004.pdf]

Supplementary Digital Content 4

Figure. Delphi survey: level of diagnostic certainty achieved by individual assessment types and combinations of these assessments when assessing whether a pain is ‘definite’, ‘probable’ or ‘possible’ neuropathic pain

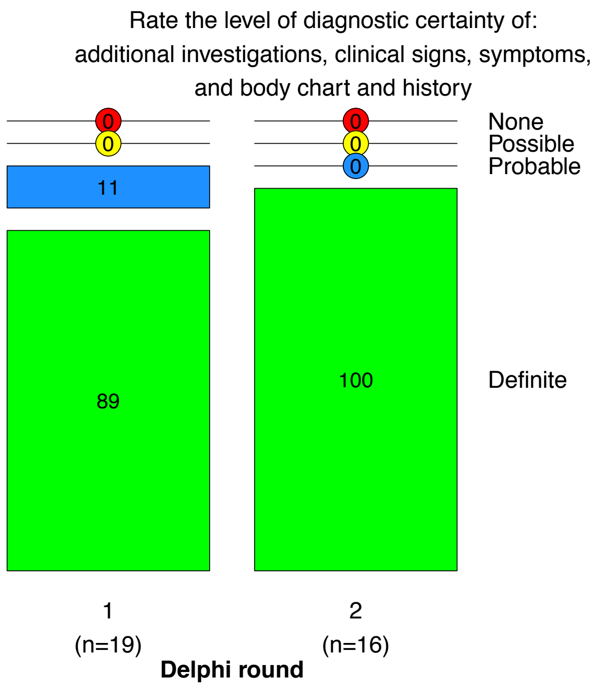
Rate the level of diagnostic certainty of:
additional investigations, clinical signs, symptoms,
and body chart and history
None
Possible
Probable
Definite
0
0
11
89
0
0
0
100
1
(n=19)
2
(n=16)
Delphi round

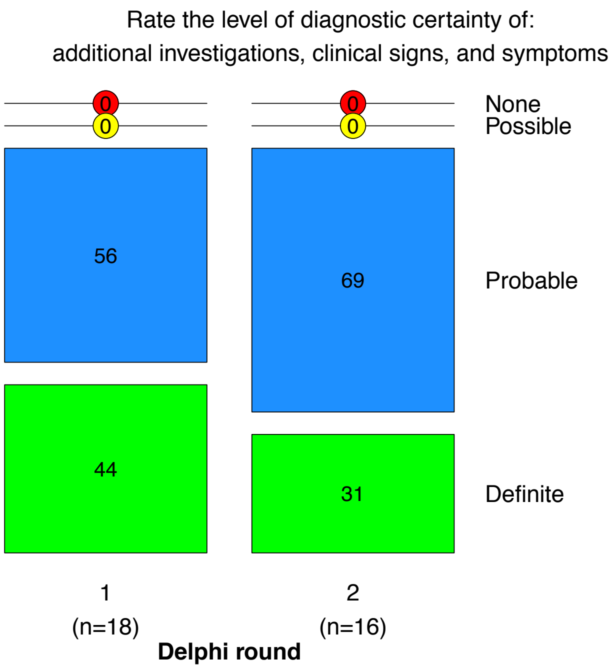
Rate the level of diagnostic certainty of:
additional investigations, clinical signs, and symptoms
None
Possible
Probable
Definite
0
0
56
44
0
0
69
31
1
(n=18)
2
(n=16)
Delphi round

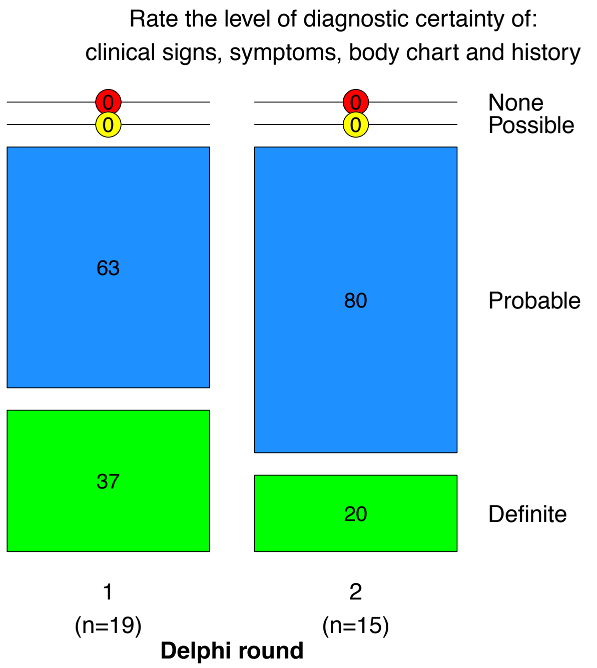
Rate the level of diagnostic certainty of:
clinical signs, symptoms, body chart and history
None
Possible
Probable
Definite
0
0
63
37
0
0
80
20
1
(n=19)
2
(n=15)
Delphi round

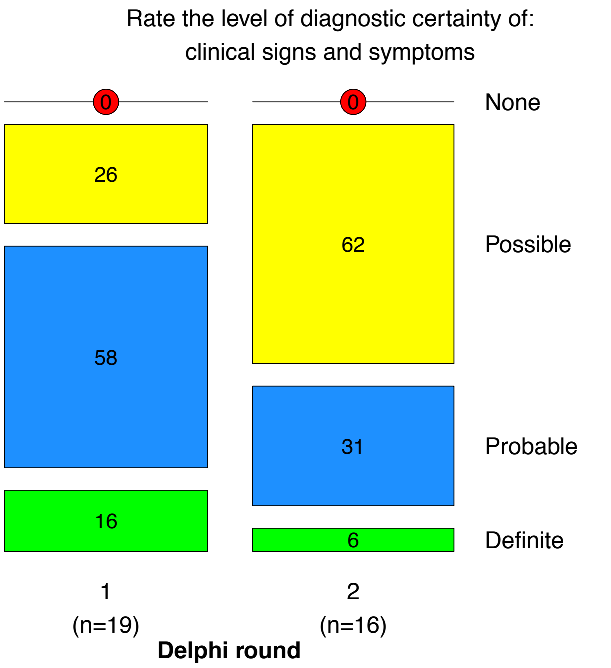
Rate the level of diagnostic certainty of:
clinical signs and symptoms
None
Possible
Probable
Definite
0
26
58
16
0
62
31
6
1
(n=19)
2
(n=16)
Delphi round

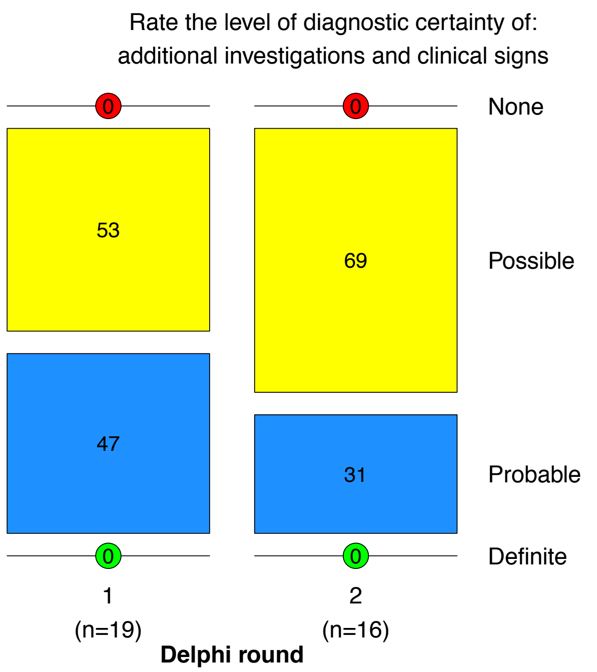
Rate the level of diagnostic certainty of:
additional investigations and clinical signs
0
0
None
53
69
Possible
47
31
Probable
0
0
Definite
1
(n=19)
2
(n=16)
Delphi round

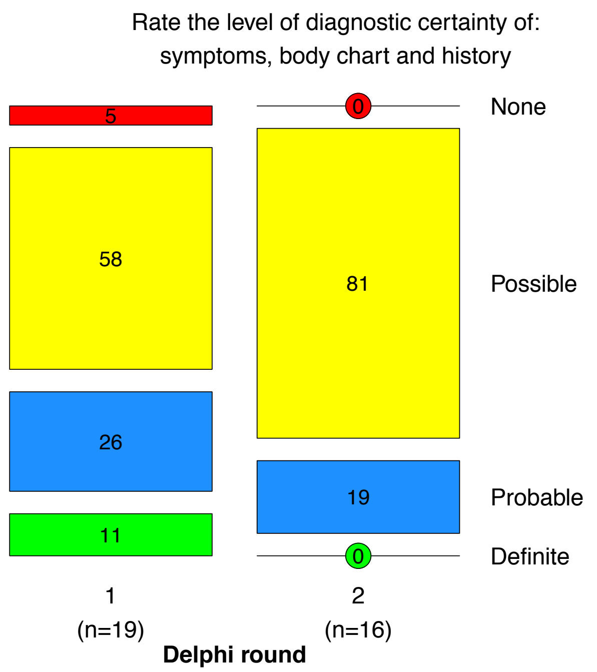
Rate the level of diagnostic certainty of:
symptoms, body chart and history
5
0
None
58
81
Possible
26
19
Probable
11
0
Definite
1
(n=19)
2
(n=16)
Delphi round

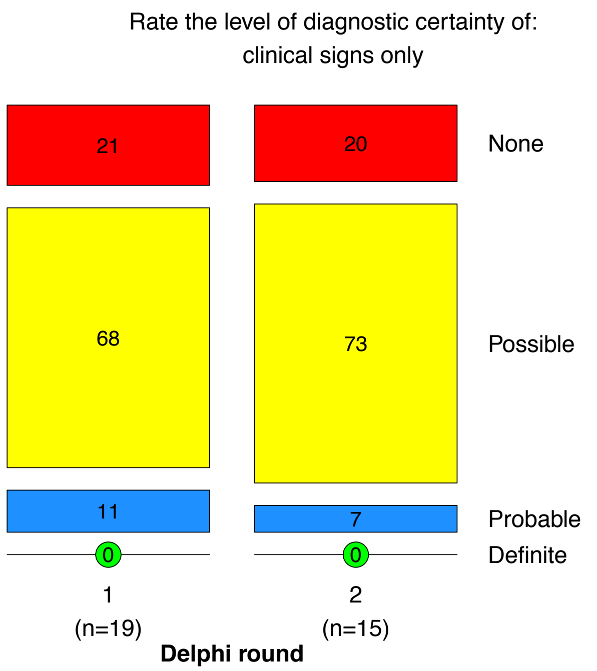
Rate the level of diagnostic certainty of:
clinical signs only
21
20
None
68
73
Possible
11
7
Probable
0
0
Definite
1
(n=19)
2
(n=15)
Delphi round

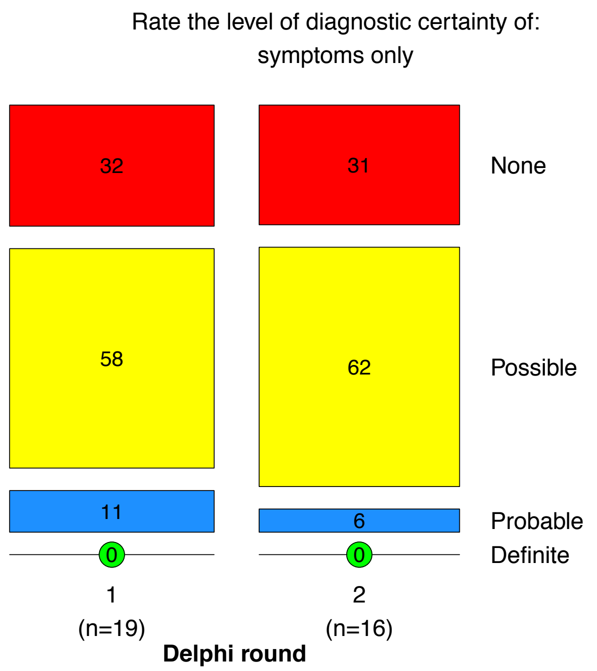
Rate the level of diagnostic certainty of:
symptoms only
32
31
None
58
62
Possible
11
6
Probable
0
0
Definite
1
(n=19)
2
(n=16)
Delphi round

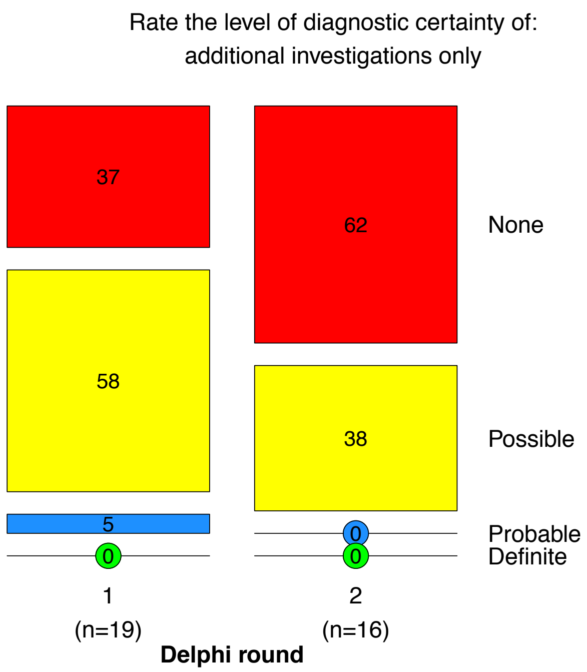
Rate the level of diagnostic certainty of:
additional investigations only
37
58
5
0
62
38
0
0
None
Possible
Probable
Definite
1
(n=19)
2
(n=16)
Delphi round
