# Supplementary material for: Neuropathic pain phenotyping by international consensus (NeuroPPIC) for genetic studies: a NeuPSIG systematic review, Delphi survey, and expert panel recommendations
Source: Pain. 2015 Oct 22;156(11):2337–53. doi: 10.1097/j.pain.0000000000000335 (PMC4747983; doi:10.1097/j.pain.0000000000000335)
Supplement: SUPPLEMENTARY MATERIAL [file jop-156-2337-s005.pdf]

Supplementary Digital Content 5

Figure. Delphi survey: level of agreement on other assessments to include when phenotyping (NP: neuropathic pain)

Other measurements

(NP: neuropathic pain)

Assessment of NP should include
psychological factors

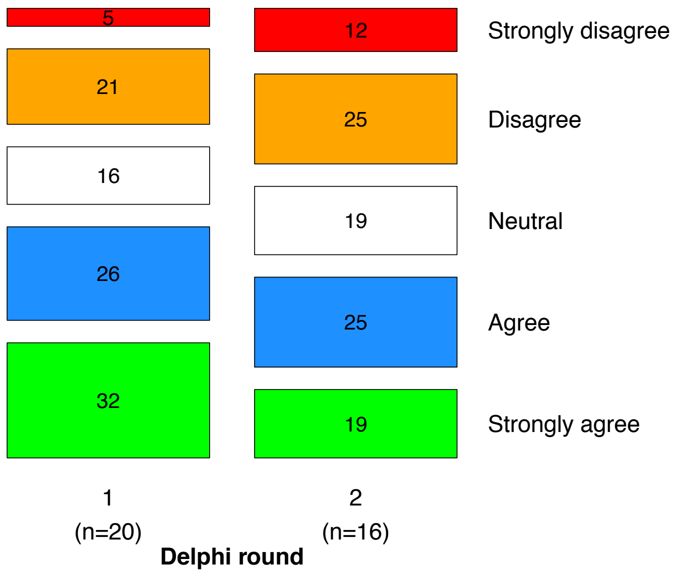

Assessment of NP should include include
quality of life measures

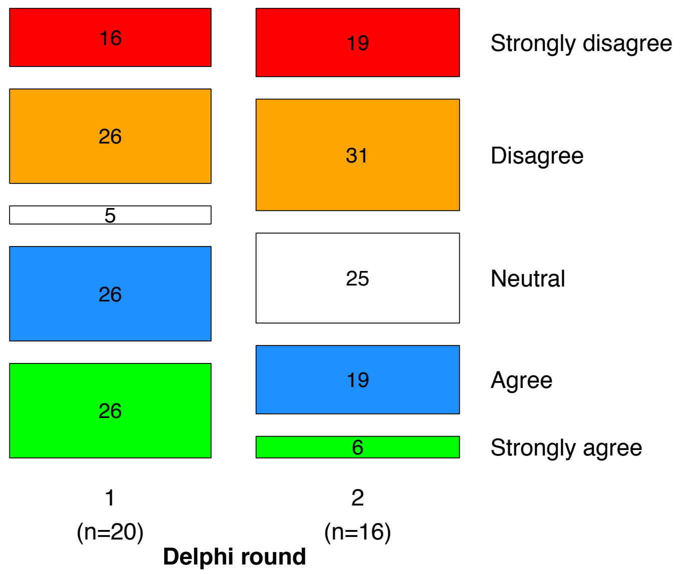

Assessment of NP
should include a history

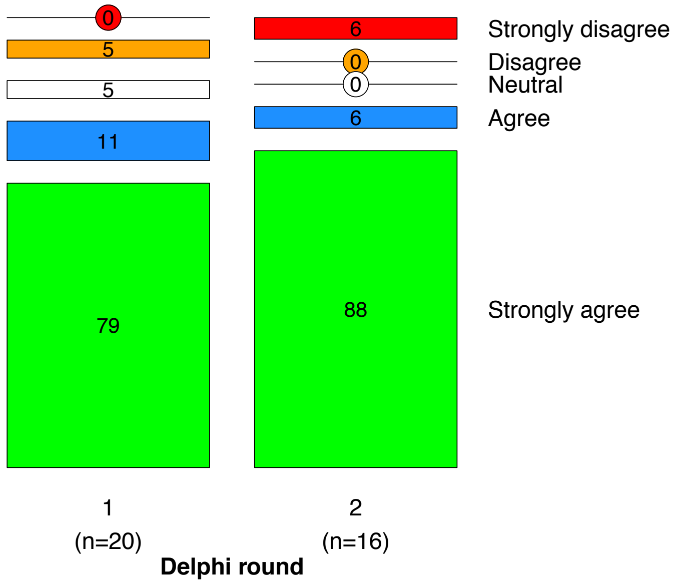

Assessment of NP
should include a body chart

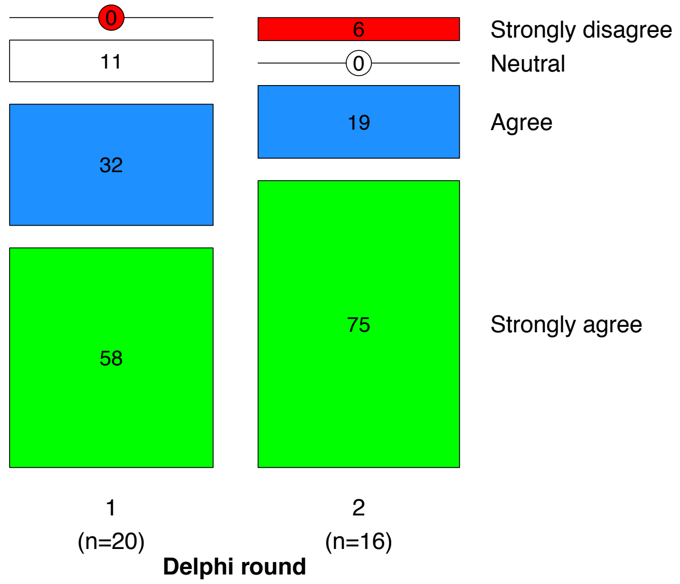

Numbers in the blocks report percent respondents choosing a category
Note: percentages may not match those presented in Table 4 because of rounding
