# Supplementary material for: Neuropathic pain phenotyping by international consensus (NeuroPPIC) for genetic studies: a NeuPSIG systematic review, Delphi survey, and expert panel recommendations
Source: Pain. 2015 Oct 22;156(11):2337–53. doi: 10.1097/j.pain.0000000000000335 (PMC4747983; doi:10.1097/j.pain.0000000000000335)
Supplement: SUPPLEMENTARY MATERIAL [file jop-156-2337-s006.pdf]

Supplementary Digital Content 6

Figure. Delphi survey: level of agreement on items to include in a patient history

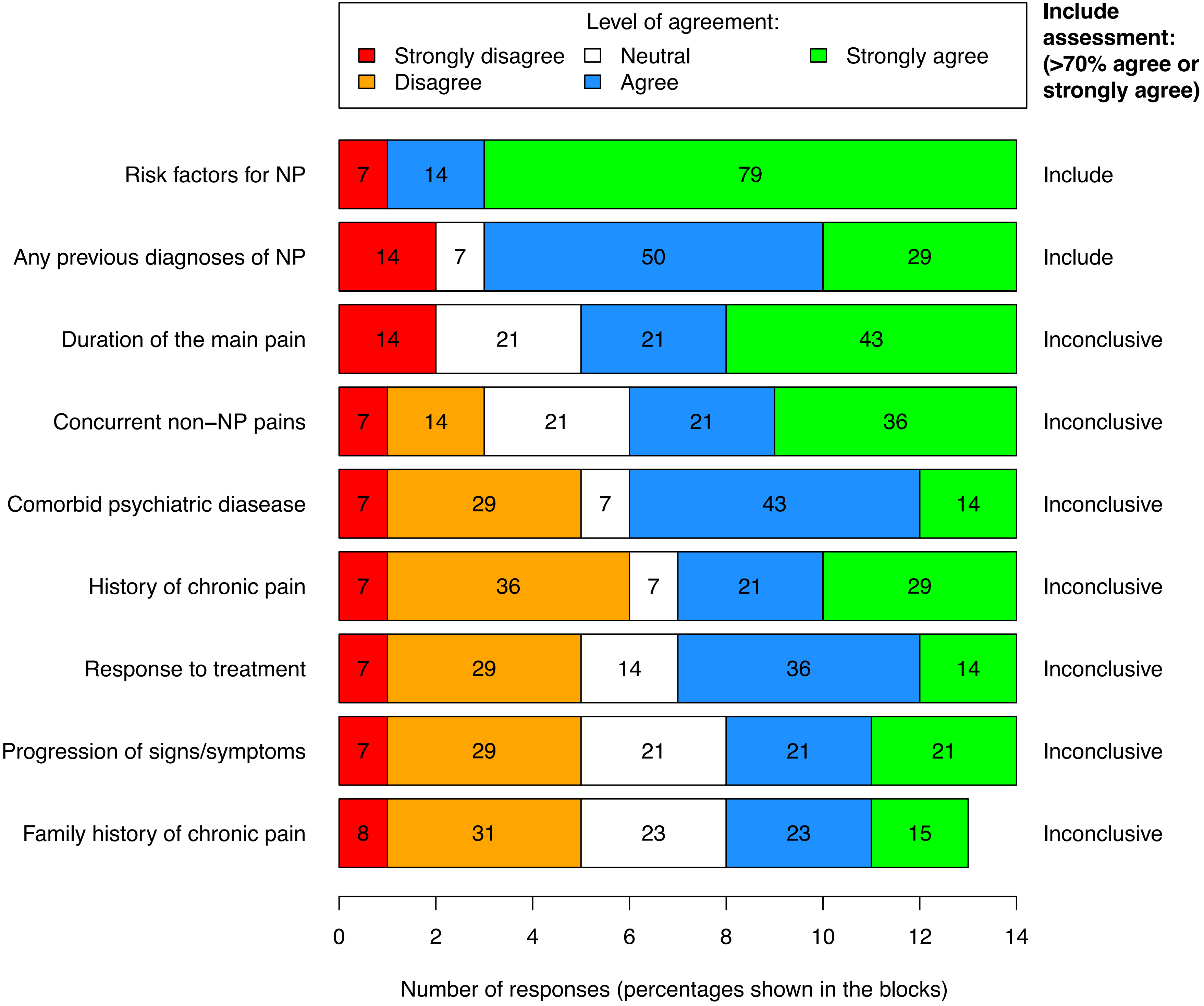
Level of agreement:
Strongly disagree
Disagree
Neutral
Agree
Strongly agree
Include assessment: (>70% agree or strongly agree)
Risk factors for NP
7
14
79
Include
Any previous diagnoses of NP
14
7
50
29
Include
Duration of the main pain
14
21
21
43
Inconclusive
Concurrent non–NP pains
7
14
21
21
36
Inconclusive
Comorbid psychiatric diasease
7
29
7
43
14
Inconclusive
History of chronic pain
7
36
7
21
29
Inconclusive
Response to treatment
7
29
14
36
14
Inconclusive
Progression of signs/symptoms
7
29
21
21
21
Inconclusive
Family history of chronic pain
8
31
23
23
15
Inconclusive
0
2
4
6
8
10
12
14
Number of responses (percentages shown in the blocks)
