# Supplementary material for: Neuropathic pain phenotyping by international consensus (NeuroPPIC) for genetic studies: a NeuPSIG systematic review, Delphi survey, and expert panel recommendations
Source: Pain. 2015 Oct 22;156(11):2337–53. doi: 10.1097/j.pain.0000000000000335 (PMC4747983; doi:10.1097/j.pain.0000000000000335)
Supplement: SUPPLEMENTARY MATERIAL [file jop-156-2337-s007.pdf]

Supplementary Digital Content 7

Box. Suggested additional phenotypes

MORE EXTENSIVE PHENOTYPING *(based on panel discussion)*
• Further demographic information<br>o Income and educational level<br>o Family history of chronic pain especially of neuropathic pain<br>o Hand dominance
• Psychological profiling<br>o Anxiety, depression, catastrophizing, somatization, post-traumatic pain disorder, personality
• Detailed pain characterisation<br>o Temporal pattern of the pain (frequency, duration, changes in intensity)<br>o Measures of unpleasantness and suffering<br>o Analysis of multiple pain sites<br>o Assessment of heat hyperalgesia<br>o In-depth assessment of pain quality and intensity (e.g., neuropathic pain symptom inventory)<br>o Interval between any inciting event and onset of pain<br>o Further questionnaire tools to increase accuracy of diagnosis<br>o Sleep disruption<br>o Past and present pain treatments and their outcomes
• Smaller cohorts, depending on feasibility, may be further phenotyped by:<br>o Clinical examination<br>o Quantitative sensory testing<br>o Intra-epidermal nerve fibre density<br>o Test-retest to confirm reliability and stability of phenotype over time
